# Beaked whale dive behavior and acoustic detection range off Louisiana using three-dimensional acoustic tracking

**DOI:** 10.1371/journal.pone.0340398

**Published:** 2026-02-04

**Authors:** Héloïse Frouin-Mouy, Kaitlin E. Frasier, John A. Hildebrand, Eric R. Snyder, Sean M. Wiggins, Lance P. Garrison, Melissa S. Soldevilla

**Affiliations:** 1 Cooperative Institute for Marine and Atmospheric Studies (CIMAS), University of Miami, Miami, Florida, United States of America; 2 Scripps Institution of Oceanography, UCSD, San Diego, California, United States of America; 3 NOAA Southeast Fisheries Science Center, Miami, Florida, United States of America; Texas A&M University, UNITED STATES OF AMERICA

## Abstract

Understanding abundance and trends of beaked whales in the heavily industrialized Gulf of America (formerly Gulf of Mexico), is critical for management but challenging with visual-based distance-sampling due to their elusive surface behavior. Acoustic-based distance-sampling methods rely on accurate modeling of detection probability as a function of distance from a recorder, requiring population-specific diving and acoustic behavior parameters, which is currently lacking for Gulf populations. To address this, we used passive acoustic tracking with two 4-channel High-Frequency Acoustic Recording Packages (HARPs) deployed off Louisiana (~1100 m depth) in 2021. Echolocation clicks detected on both recorders were localized in 3D to characterize acoustic and diving behavior. These data informed a Monte Carlo cue-based simulation to estimate the probability of detection by a near-seafloor single-sensor HARP. A trial-based approach also estimated detection probability as a function of range to a single-channel sensor deployed at the site. Results show species-specific differences. Goose-beaked whales (*Ziphius cavirostris*), were detected for longer periods during foraging dives (n = 24 dives, mean: 20.5 min; range: 7–42) compared with Blainville’s (*Mesoplodon densirostris*, n = 2 dives, 13.6 min; 11–16) and Gervais’ (*Mesoplodon europaeus*, n = 24 dives, 12.7 min; 7–19) beaked whales. Maximum dive depths also differed, with some goose-beaked whales foraging at or near the seafloor. Descent and ascent rates were similar within species but differed among them (1.34/1.40 m/s for goose-beaked and 1.15/1.19 m/s for Gervais’ beaked whales). Source level and broadband directivity index were estimated at 225 dB_pp_ re 1 μPa-1m and 26 dB for goose-beaked whales, and 218 dB_pp_ re 1 μPa-1m and 20 dB for Gervais’ beaked whales. Estimates were not possible for Blainville’s beaked whales due to limited data. In both the Monte Carlo simulation and trial-based approach, detection probability declined sharply with ranges, reflecting the highly directional beam of beaked whale echolocation clicks.

## Introduction

Beaked whales, an elusive group of deep-diving marine mammals, are difficult to assess visually due to their lengthy deep foraging dives [1]. These dives can last more than two hours and reach depths of up to 3000 m [2], and surface intervals during which sightings can occur are short, typically between 2 and 8 min [2–4]. Passive acoustic recording methods that detect sounds produced by animals echolocating during dives provide an alternative approach for population assessment. Beaked whales were one of the first groups for which acoustic density estimation was proposed [5]. Beaked whales tend to be found in small groups [6], and during deep foraging dives, they produce species-specific frequency-modulated echolocation pulses [7] at relatively steady rates while searching for fish and cephalopod prey [8,9], making them good candidates for species occurrence and density estimation using passive acoustics [10,11].

Estimating spatial population density from detected echolocation counts requires, among other factors, determining the area around each passive acoustic receiver where echolocations can be detected. Distance-sampling methods used for acoustic density estimation require estimating a species-specific “detection function”, or the probability of detecting an acoustic signal (cue) given an animal’s distance from an acoustic recorder [10]. The preferred way to estimate the detection function is to track a set of freely swimming animals near the acoustic recorder, either using an array of fixed passive acoustic sensors and location-sensing tags [5] or by observing them from an elevated vantage point [12,13]. However, these methods are costly and potentially unfeasible in offshore locations. Alternatively, the detection function can be estimated by simulating clicks at a set of distances [14,15]. Knowledge of beaked whale source levels and characteristics of their echolocation beam pattern, as well as detailed population-specific information on subsurface dive behaviors such as subsurface group behaviors, individual and group orientations, and the influence of bathymetry and acoustic behavior, is critical for accurate detection probability estimation. Distributions of these parameters can be used in a Monte Carlo simulation to estimate the probability of detecting beaked whale clicks [16].

Several species of beaked whales are known to be present in the Gulf of America (formerly the Gulf of Mexico; hereafter the Gulf), including: goose-beaked (*Ziphius cavirostris*), Gervais’ (*Mesoplodon europaeus*), Blainville’s (*M. densirostris*), BWG (unidentified species of beaked whale in the Gulf), and possibly Sowerby’s (*M. bidens*) beaked whales [14,17]. Many of the behavioral and sound production parameters used for detection function simulations have been determined from tagging studies [18]. Though effective, tagging studies have been successfully applied to only a small subset of the 24 recognized species of beaked whales in a limited number of areas because tagging these elusive offshore species is particularly challenging. Furthermore, the difficulty of attaching tags limits the number of individuals per species that can be studied with this approach. Gervais’ beaked whales have not been tagged as of 2025, to our knowledge, and only one published study has reported their click characteristics with visual species confirmation [19]. Due to the lack of tag data for goose-beaked, Gervais’ and Blainville’s beaked whales in the Gulf, behavioral parameters from other regions and in some cases from other species have been used as substitutes in model-based beaked whale detection probability estimation [14,16]. However, both goose-beaked and Blainville’s beaked whales exhibit regional variation in diving [e.g., 1,2,20] and acoustic [e.g., click production rates; 21] behaviors. Furthermore, diving behavior is not consistent across all beaked whale species with Sowerby’s beaked whales (*Mesoplodon bidens*) showing markedly different foraging patterns compared to goose-beaked and Blainville’s beaked whales [22].

Passive acoustic tracking methods, which use time difference of arrival (TDOA) of signals across multiple acoustic receivers to estimate source locations, provide an alternative approach for obtaining dive and acoustic behavior metrics for deep-diving whales at locations of interest. This method has been applied to beaked whales using towed linear hydrophone arrays [23], drifting acoustic spar buoy recorders [DASBRs; 24], distributed bottom-mounted hydrophones in conjunction with a digital acoustic recording tag [25], and bottom-mounted hydrophone arrays [26–29]. Passive acoustic tracking relies on active sound production by animals; therefore, animals which are not acoustically active cannot be localized. Additionally, species that produce highly directional signals like beaked whales are less likely to be detected when they are oriented away from sensors in the array; therefore, capturing full dive cycles can be rare depending on the sensor configuration [e.g., 26]. However, passive acoustic tracking can help achieve much larger sample sizes compared to approaches using telemetry tags. It also provides the ability to characterize key aspects of their echolocation clicks, including source levels [26], and allows estimation of directionality [30], depth distribution [31], range [24], and minimum group size [26]. In a towed hydrophone array study, group size was generally found to be underestimated compared to visual counts, likely because clicks produced at similar bearing angles caused multiple individuals to be merged when assigning clicks to individuals [32]. In our study, we used a tetrahedral array, which helped to resolve this issue. Tagging studies have shown that goose-beaked and Blainville’s beaked whales forage along different headings while maintaining tight group cohesion, typically remaining tens to hundreds of meters apart [33,34]. These distances are still within the detection range of our tracking HARPs, allowing us to capture most, if not all, individuals within a group.

In this study, a bottom-moored array was deployed for six months in the north-central Gulf to acoustically track deep-diving beaked whales. The goal of this project is to improve beaked whale density estimates in the Gulf by more accurately estimating species-specific detection probabilities by incorporating population-appropriate signal and behavioral parameters. We quantify Gulf specific-acoustic and dive parameters required for estimating cue detection probabilities for the three most commonly occurring Gulf beaked whale species. The regionally-appropriate parameters derived from acoustic tracking are used to improve simulations of beaked whale subsurface behavior in the Gulf. Horizontal range, elevation angle and received level distributions from localized encounters are compared to species-specific model predictions. Finally, using tracking data to determine where and when clicks are produced by beaked whales in the vicinity of a single-sensor acoustic recorder, we estimate click detection probabilities and compare them to the predictions of species-specific Monte Carlo simulations. Results presented for three Gulf beaked whale species include the first detailed description of the diving behavior and water column use of Gervais’ beaked whales.

## Materials and methods

### Ethic statement

This study collected acoustic recordings containing beaked whale echolocation clicks using bottom-mounted recorders, a non-invasive field technique to remotely survey the sounds produced by living marine resources that has little potential to adversely affect the environment or interfere with organisms or habitat. This activity posed no potential for significant environmental effects and therefore did not require any special permits.

### Data collection

**Fig 1:**
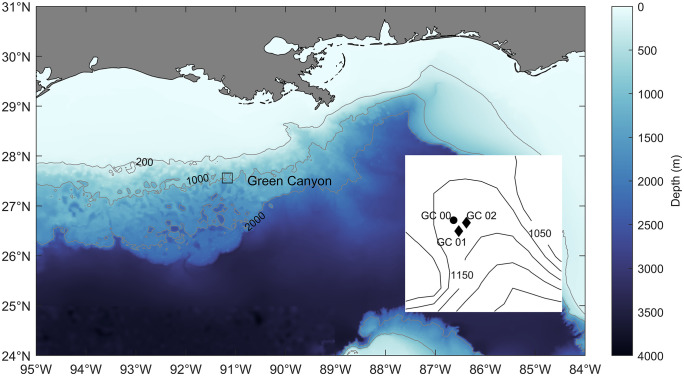
Gulf study site near Green Canyon (GC) where the tracking HARP array was deployed for acoustic tracking of beaked whales and recorded for ~ 6 months. Bathymetric contours at 200 m, 1000 m and 2000 m are light gray. Inset: Two multi-channel tracking HARPs (diamond symbols) were deployed at GC 01 and GC 02, and a single-channel HARP (circle symbol) was deployed at GC 00. Bathymetric contours at 1050, 1100, 1150, 1200, and 1250 m are illustrated. The map was generated with Matlab using the ETOPO2 data [37].

From August 2021 to May 2022, an array of one single-hydrophone and two tracking High-frequency Acoustic Recording Packages (HARPs) were deployed at a site near Green Canyon (GC, Fig 1), an area monitored since 2010 and known for the presence of beaked whales, including goose-beaked, Gervais’ and Blainville’s beaked whales [14]. HARPs are bottom-mounted acoustic recorders equipped with hydrophone(s), data logger, battery power supply, ballast weight, acoustic release system, and flotation device [35]. Acoustic recordings were converted to sound pressure levels using hydrophone calibrations conducted at Scripps Institution of Oceanography and the U.S. Navy’s Transducer Evaluation Center in San Diego, California. Tracking HARPs are equipped with four calibrated, time-synchronized hydrophones positioned in a small-aperture tetrahedral array with a ~ 1 m sensor spacing [29]. The 4-element array was mounted on top of a fiberglass mast with the top hydrophone positioned approximately 6 m above the seafloor. In addition to the two tracking HARPs, a third single-channel HARP mooring was deployed in the vicinity and provided recordings for the trial-based approach. The single-hydrophone HARP (GC 00, Table 1) recorded continuously at 200 kHz sample rate with the sensor approximately 10 m above the seafloor from August 20, 2021, to May 5, 2022. The two tracking HARPs (GC 01 and GC 02) were deployed 772 m apart and recorded all four hydrophones continuously at 100 kHz sample rate from September 7, 2021, to March 25^th^ and 26^th^, 2022 respectively (Table 1, Fig 1). GC 00 was deployed at distances of 744 m and 824 m from GC 01 and GC 02, respectively. Instrument positions, after settling on the seafloor, were localized by applying a least-squares inverse to the two-way travel times from the support vessel to the HARPs and back while the ship circumnavigated the recorders and sending 11 kHz interrogation pings from a towed transducer with known global positioning system (GPS) locations [29,36].

**Table 1:** HARP deployment periods and locations.

Site ID	Number of channels	Sample rate (kHz)	Start Date MM/DD/YY	End Date MM/DD/YY	Recording Duration (Days)	Deployment Long. W	Deployment Lat. N	Deployment Depth (m)
GC 00	1	200	08/20/21	05/05/22	258	91.16930	27.55760	1103
GC 01	4	100	09/07/21	03/26/22	200	91.16586	27.55165	1131
GC 02	4	100	09/07/21	03/25/22	199	91.16096	27.55706	1114

To determine the relative positions of hydrophone sensors within the small-aperture arrays, TDOAs were calculated using the propeller cavitation noise produced by the support vessel used to deploy and localize the instruments. The propeller cavitation noise was bandpass-filtered and cross-correlated in one-second bins as the ship circled and crossed over each array. The TDOAs were then used in a matrix inversion to estimate the relative positions of hydrophones within each array [28]. Information provided by the manufacturer (Seascan Inc.) suggested that the HARPs’ clock typically drifted by approximately 0.6 s over a year-long deployment.

### Acoustic detection and classification of beaked whale sounds

Beaked whales produce two types of echolocation clicks while diving: frequency-upswept echolocation clicks used for prey detection and buzz clicks used in the final stages of prey capture [38,39]. Buzz clicks have a 10–20 dB reduction in source level compared to echolocation clicks, making them more difficult to detect and localize [39,40], and were therefore omitted from our study. Two key parameters used for identifying beaked whale species are the inter-click interval (ICI) and peak frequency [7]. Goose-beaked whales have the longest ICI among the three Gulf beaked whale species, with a typical duration around 500 ms [14], differing from its ICI in other regions where its modal value ranged between 337–465 ms [7,41–44]. In the Gulf, Gervais’ beaked whale has a modal ICI around 290 ms [14], slightly lower than Blainville’s beaked whale modal ICI at 320 ms [14], both similar to their counterparts in other regions; Gervais’ beaked whale [19,44], Blainville’s beaked whale [7,38,39]. Goose-beaked whales have 2 distinct minor spectral peaks in their clicks at 18–19 kHz and 22–24 kHz [24,43–46], and a broad peak between 25 and 50 kHz [7,14]. Gervais’ beaked whales have a small spectral peak at ~21 kHz, and a broad peak between 30 and 55 kHz [7,14]. Blainville’s beaked whales have a small spectral peak at 23 kHz, and a broad peak skewed toward lower frequencies than the other two, with the majority of the energy between about 25–40 kHz [7,14]. The upper frequency ranges observed in uncontrolled ocean studies are influenced by propagation loss and differ from near field estimates [26].

The first phase of data analysis focused on finding all beaked whale acoustic encounters within the dataset. Beaked whale echolocation clicks were automatically detected and manually classified in a two-step approach, using custom software programs based in MATLAB (Mathworks, Natick, MA). In the first step, potential toothed whale echolocation clicks were detected automatically using a generic pulse detector, the publicly-available *SPICE-Detector* remora package [47] in *Triton* [35]. The detection algorithm used a fifth-order Butterworth filter with a high-pass range from 5 to 100 kHz (or 50 kHz for the tracking HARPs) to eliminate low-frequency noise. Signals were identified as odontocete echolocation clicks if the energy exceeded a specific received level (RL) threshold and if the signals’ peak frequencies and durations fell within typical ranges for the odontocete echolocation clicks. A minimum RL of 118 dB peak-to-peak (pp) re 1 μPa^2^ was imposed to ensure a consistent detection range around the recorder [48]. Signals were retained if they had durations between 30 and 1200 μs and peak frequencies between 5 and 100 kHz, a broad range designed to retain all potential odontocete echolocation signals above the minimum received level threshold for subsequent classification [47].

In the second step, following automatic detection, acoustic events (bouts of detections) were manually classified by species based on the ICI and the overall shape of the mean spectra across all clicks within each encounter. Encounters were identified as periods containing beaked whale clicks, bounded before and after periods greater than 30 minutes [approximated period of vocal activity in beaked whales; 4] without beaked whale clicks. All detections were manually verified and classified as beaked whale species [based on 14] using *DetEdit* [49], an open-source, MATLAB-based detection labeling tool. *DetEdit* provides interactive displays to facilitate comparison of detections, offering information on features such as individual or averaged waveforms, spectra of selected detections, ICIs, and long-term spectrograms to contextualize detections more broadly. Within each encounter, false detections (e.g., signals from vessels, sonar, sperm whales or delphinids) were manually identified and discarded.

### Three-dimensional localization

The second phase of data analysis focused on reconstructing individual tracks of diving whales (3D localizations) from echolocation pulses from goose-beaked, Gervais’, and Blainville’s beaked whales detected on both tracking recorders at GC using a toolkit called *Where’s Whaledo* [28]. This MATLAB package is designed to annotate click detections and reconstruct tracks from acoustic array recordings using a combination of automated localization methods and manual annotation of graphical data. *Where’s Whaledo* is specifically designed to accommodate recordings that pair two volumetric small-aperture arrays (e.g., tracking HARPs) for acoustic localization. By measuring the TDOA between the hydrophones within one small-aperture array, the Direction Of Arrival (DOA) of the sound can be estimated as an azimuth and elevation angle to the animal. When localizing with two DOAs (one from each tracking HARP), the source location (in 3D) is estimated as the point along one DOA where the distance to any point along the second DOA line is minimized.

*Where’s Whaledo* was used to localize whales within encounters identified in the first phase. A generic beaked-whale-specific detector within *Where’s Whaledo* was run to detect all beaked whale echolocation clicks within the previously identified encounters (adding a 15 min buffer before and after each encounter to ensure no clicks were missed). This detector used a fourth-order, zero-phase, high-pass elliptic filter with a cutoff frequency of 20 kHz, a peak-to-peak stop-band ripple of 0.1 dB, and a minimum stop-band attenuation of 40 dB. The acoustic waveform was cross-correlated around each detection across other receivers in the array to determine the small-aperture TDOA. The TDOA was subsequently converted to azimuth and elevation angles. Azimuth is defined as the counterclockwise horizontal angle when viewed from above, where East is 0°, and North is 90°. Elevation angle is defined as the vertical angle, where 0° is down toward the center of the earth, 90° is horizontal in the plane of the tetrahedral hydrophone array, and 180° is upward toward the sea surface.

To aid in data curation, a graphical user interface (GUI) tool called *brushDOA* was used to remove false detections, identify unique sources (i.e., individual whales), and associate detections across the two small-aperture arrays. This interface features six plots (S1 Fig): azimuth vs. time, elevation vs. time, and azimuth vs. elevation for both 4-channel arrays (tracking HARPs). Users can interactively remove data points from the dataset or assign color labels (representing individual whales) to detections from the same source. By observing gradual changes in both azimuth and elevation angles, it is possible to identify collections of detections originating from a single source (as in the example of three goose-beaked whale tracks in S1 Fig). Estimates of beaked whale minimum group size were derived from acoustic encounters as the total number of distinct tracks visible in *brushDOA* during each encounter.

After assigning azimuth and elevation points to individual whale tracks on one of the arrays, *Click-Train Correlation* (CTC) was used to associate detections across the two arrays [28,50]. The CTC method matches patterns of clicks by aligning a set of detected clicks in a 30-second window of time on different instruments to determine which clicks originated from the same source. The time delay required to align the click trains also produces an estimate of the TDOA (*t*). Detections in the unlabeled array that align with labeled detections (delayed by *t*) are assumed to originate from the same source and are labeled accordingly.

**Fig 2:**
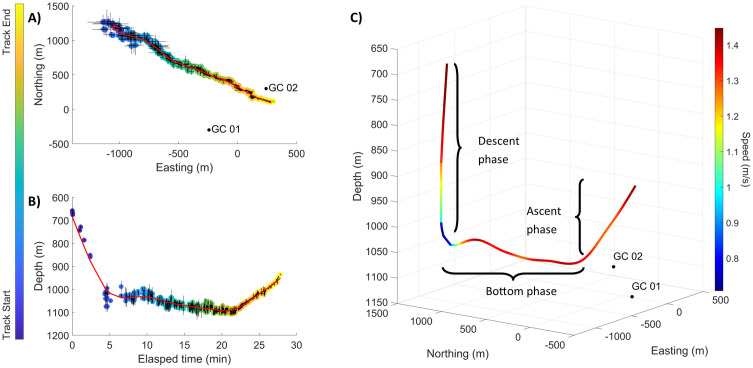
Example of an acoustically localized track estimated over 28-min of a dive for a goose-beaked whale detected on both tracking HARPs (black dots; GC 01 and GC 02) on Dec 1, 2021. **(A)** A map view of individual click 2D localizations (circle markers’ color represents elapsed time, with gray error bars) and the smoothed estimated spatial track (red line). **(B)** The estimated depth for individual click localizations (circle marker’s color represents time, with gray error bars), and the smoothed estimated depth track over time (red line) shows descent, bottom, and ascent phases indicating this track likely captured the complete acoustic portion of this goose-beaked whale dive. **(C)** A 3-D reconstruction of the dive including swim speed estimates (color bar indicates speed), showing the whale slowing down as it approaches the bottom phase of the dive and the different dive phases (descent, bottom and ascent).

The labeled detections across both tracking arrays were localized in three dimensions (X, Y, Z) by finding the closest point of intersection between the two DOA lines. The confidence intervals for this method of localization were obtained using the jackknife variance estimator, taking advantage of the fact that six TDOAs can be computed across the four hydrophone channels, but a DOA can be estimated from as few as three TDOAs. Using the jackknife method, one TDOA and its associated receiver pair was removed from the DOA estimation, and a new DOA was estimated using the remaining five TDOAs. A new whale location was estimated using the intersection point of the newly obtained DOA and the DOA of the other array. This was repeated for each receiver pair, removing one TDOA and localizing with the remaining 11, until 12 different whale location estimates were produced. The variance of these location estimates was determined and used in the inverse Student’s T distribution to estimate the 95% confidence intervals. A spline smoothing function (smoothing parameter = 1 × 10^−8^) was applied to X, Y, and Z positions over time, yielding 3D dive tracks for individual whales (Fig 2). The uncertainties in the assumed sound speed and in instrument positions were incorporated into the overall uncertainty of the localization estimates following Snyder et al. [28].

To ensure only the highest quality tracks were used for estimating dive behavior parameters for density estimation, some beaked whale tracks were removed before conducting the remaining analyses. Tracks were removed if 1) they represented groups of animals whose detection angles could not be unambiguously discriminated to each individual, 2) they contained too few detections (fewer than 100 clicks per HARP) making reliable localization or refined dive profiling infeasible, or 3) their duration was too short (less than 7 min, selected based on a review of the usable tracks in this dataset). Given the limited knowledge about Blainville’s beaked whales in this region, we included the only two available tracks in our analysis, recognizing that some information, with appropriate caveats, is better than none. An ANOVA followed by Tukey-Kramer’s HSD post hoc test was used to compare dive and acoustic parameters among beaked whale species.

### Diving behavior parameter estimation

Parameter distributions for four dive behavior features that are required for detection probability simulations can be empirically measured from the tracks: dive depth (mean and standard error), dive altitude above the seafloor (mean and standard error), depth at the start of the clicking period (mean and standard error), and descent pitch angle (mean and standard error). The duration of each dive track was calculated as the time between the first and last localized clicks associated with the whale; dive tracks ended either when the whale stopped echolocating or when clicks were no longer detected. Each dive track was divided into up to three components: descent, foraging, and ascent, when any of these components were present. Tracks did not necessarily represent complete dives; therefore, they did not always have all three components. We defined the descent portion of a dive as the period from the first detection of echolocation clicks associated with decreasing depth (total difference > 200 m) until the whale reaches a depth and stabilizes (depth variance was limited to 50 m for at least 5 min). If this component existed within a track, it was always the first segment. The ascent portion of the dive was defined as the extent from the first deep position associated with consistently increasing depth (total difference > 80 m) until the whale stopped clicking or was not detected anymore. If this component existed within a track, it was always the last segment. The foraging portion of the dive was defined as the bottom part of the track occurring between the descent and ascent portions, even if the whale was not at the seafloor or stabilized to a specific depth. This component, if present, could be the first, middle or final segment of a track. Swim speed was estimated for each dive portion (descent, foraging and/or ascent) by dividing the sum of covered smoothed distance (in 3D) between two localizations by the sum of elapsed time between two localizations. Descent and ascent pitch angles were estimated by arcsin(∆ depth/ covered distance). Track durations and swim speeds were calculated for comparison with previously published values.

### Acoustic behavior parameter estimation

Parameter distributions for three acoustic behavior features that are required for detection probability simulations can be empirically measured from the acoustically localized click detections: peak frequency (mean and standard deviation), apparent source level (mean and standard deviation), and directionality index (mean). Click peak-to-peak apparent source levels (ASL_pp_) were estimated assuming spherical spreading and frequency dependent absorption as


$${ASL}_{pp}={RL}_{pp}+\text{2}\text{0}{log}_{\text{1}\text{0}}(r)+\alpha \left(f_{p}\right)*\frac{r}{\text{1}\text{0}\text{0}\text{0}}$$ (1)

where RL is the peak-to-peak received level, *r* is the estimated distance (in meters) between the source (whale) and the receiver (one hydrophone in the tracking HARP), and α(*f*_*p*_) is the frequency dependent absorption coefficient in dB/km [51]. The absorption coefficient [α; 52], was computed based on the peak frequency (*f*_*p*_) for each beaked whale click and *in situ* CTD measurements taken during HARP deployments, using a temperature of 4.7 °C, a salinity of 35 ppt, a pH of 8, and a depth of 1.1 km (~ seafloor depth at HARP locations). At this depth, temperature, salinity and pH are expected to be stable throughout the year, so values obtained during HARP deployments were assumed to be constant across seasons. Only localized clicks in high quality tracks were used.

The effect of directionality of beaked whale click transmission on received levels was estimated by associating the ASL_pp_ of each click with an estimate of the whale’s orientation with respect to the receiver. The orientation was estimated as the angle measured between the 3D vector (whale’s body orientation) connecting the (smoothed) whale’s position at *t* (time of click) and *t* + 1 (time of the next click), and the 3D vector (whale to receiver) connecting the (smoothed) whale’s position at *t* with the HARP from which the ASL_pp_ was estimated. This 3D off-axis angle, *γ*, ranges from 0 (looking forward along the acoustic axis) through 90° (perpendicular to the acoustic axis) to 180° (looking backward along the acoustic axis) [26]. The median values for each off-axis angle bin (bin size: 2°) were calculated. The directivity index (DI), which represents the sharpness of the beam or main lobe in a receiving or transmitting beam pattern, was used to estimate the off-axis amplitude loss using the piston model, following Zimmer et al. [53]. Values of source level (SL_pp_) and DI for goose-beaked and Gervais’ beaked whales were estimated by fitting a circular piston model to the empirical median ASL distributions, over the 0–90 degree angle range, using preliminary model parameters for DI and SL obtained from beaked whales in other regions. SL_pp_ and DI were not estimated for Blainville’s beaked whales due to limited data.

### Detection probability

Two approaches were used to estimate click detection probabilities for each of the three species: 1) a detectability simulation and 2) an empirical case study (trial-based approach). The first approach, cue-based detection probability simulation, was designed to estimate the probability of a near-seafloor single sensor detecting individual clicks. This approach used two nested loops within a Monte Carlo framework, modeled after Küsel et al. [15] and refined by Frasier et al. [36]. These simulations took into account acoustic and behavioral parameters, including minimum click amplitude detection thresholds, DI, echolocation click peak frequency content (rounded to the nearest even number) and distance-dependent attenuation, animal dive depths, and ascent and descent rates. To introduce uncertainty and variability, across model iterations, a bootstrapping procedure was implemented. For each species, within each of 500 iterations (N) of the outer loop, 100,000 animat models were simulated, with input parameters (P) defined with a mean (µ_PN_) and standard deviation (σ_PN_) drawn from uniform distributions. These input parameters were associated with the acoustic characteristics of the signal and animal behavior, and their limits were derived from both this study (Table 2) and/or relevant literature where missing (Table 3). The simulated sensor was assumed to be located at the same depth and near the position of the deployed tracking HARPs. Within a single model iteration, 10,000 source positions were randomly distributed across a circular area with a 4 km radius in the horizontal plane around the sensor. Each source (representing an animal) was assigned a depth based on a log-normal probability distribution, with parameters derived from the outer-loop bootstrapping procedure. Sources were also assigned on-axis source levels and beam directivities, drawn from distributions informed by values reported in the literature and this study. Beam directivities were used to generate three-dimensional beam patterns following a piston model [53,54] and incorporated into transmission loss calculations. Predicted received levels (RL) at the sensor were calculated for clicks produced at each modeled source position, using the assigned source parameters and model-based transmission loss estimates at the click peak frequency. Clicks with RL exceeding 118 dB_pp_ were deemed detectable, with this threshold chosen to reduce the impact of variable background noise levels on detection counts. Detection probability (*Pdet*) for each simulation was determined as the ratio of detected clicks to the total number of simulated clicks.

**Table 2:** Species-specific dive statistics (number of individuals, mean ± standard deviation, minimum and maximum) derived from tracks. Summary statistics for each track and each species are provided in Tables S1–S3.

	Duration (min)	Depth (m)			Speed (m/s)			Pitch angle (°)	
Species		Minimum	Maximum	Mean	Descent	Bottom	Ascent	Descent	Ascent
**Goose-beaked whale**									
*n*	24	24	24	24	9	24	3	9	3
Mean ± sd	20.5 ± 9.3	808 ± 235	1078 ± 71	983 ± 95	1.34 ± 0.15	0.99 ± 0.32	1.40 ± 0.16	68.5 ± 8.83	26.1 ± 8.95
Min – max	7.0–42.1	425–1162	888–1208	838–1172	1.14–1.59	0.29–1.97	1.24–1.62	51.6–84.3	14.4–36.2
**Gervais’ beaked whale**									
*n*	24	24	24	24	3	24	3	3	3
Mean ± sd	12.7 ± 3.6	801 ± 83	910 ± 71	865 ± 71	1.15 ± 0.16	1.13 ± 0.28	1.19 ± 0.06	35.7 ± 8.51	12.1 ± 4.68
Min – max	7.1–19.2	657–1000	786–1062	770–1026	0.93–1.29	0.56–1.83	1.11–1.26	23.7–42.5	7.55–18.5
**Blainville’s beaked whale**									
*n*	2	2	2	2	1	2	NA	1	NA
Mean ± sd	13.6 ± 2.7	711 ± 91	861 ± 21	795 ± 36	1.14	1.09 ± 0.36	NA	36.3	NA
Min – max	10.8–16.3	620–801	839–882	759–831	NA	0.73–1.44	NA	NA	NA

**Table 3:** Monte Carlo detectability simulation parameters by species. Assumed variable values are in italics. References are listed as superscripts: a: Shaffer et al. 2013; b: Tyack et al. 2006; c: Zimmer et al. 2005; d: Hildebrand et al. 2015. * This study.

Simulation parameter ranges/ Species	Goose-beaked whale	Gervais’ beaked whale	Blainville’s beaked whale
*Acoustic parameters*			
Source Level Mean (dBpp)	223–228*	215–220*	*215–220* ^a^
Source Level Std. Dev. (dBpp)	1–4	1–2	2–5
Peak Frequency (kHz)	40*	40*	34*
Minimum off-axis amplitude loss (dBpp) – Side	*28–32* ^c^	*28–32* ^c^	*28–32* ^c^
Minimum off-axis amplitude loss (dBpp) – Back	*28–32* ^c^	38–42*	38–42*
Directivity Index	24–28*	18–22*	*21–25* ^a^
*Dive parameters*			
Depth at Start of Clicking Mean (m)	450–460*	*420–430* ^b^	*420–430* ^b^
Depth at Start of Clicking Std. Dev. (m)	60–80	20–30	20–30
Dive Depth Mean (m)	980–990*	860–870*	790–800*
Dive Depth Std. Dev. (m)	90–100	60–80	30–50
Dive Altitude Mean (m)	100–110*	200–300*	300–400*
Dive Altitude Std. Dev. (m)	50–60	50–100	50–100
Dive Depth Max (m)	3,000	3,000	3,000
Descent Angle Mean (degrees)	70–75*	35–40*	*72–77* ^b^
Descent Angle Std. Dev. (degrees)	5–10	5–10	5–10
Maximum model radius (km)	4	4	4
Number of points simulated	100,000	100,000	100,000

The second approach (trial-based approach) used beaked whale 3D tracks from this study to estimate whether an animal at a given horizontal range and orientation (off-axis) was detected on an independent sensor (HARP GC 00). For each “complete track”, between the first and final localized clicks, linear interpolation was used to obtain the position of the tracked whale at each second. For each of the three beaked whale species, an indicator of detection (1 – detected; 0 – not detected) was generated for each second of the complete tracks, if at least one click, corresponding to the tracked beaked whale species, was received at HARP GC 00 (single sensor) during this same second. Then, the horizontal distance and off-axis angle between the whale and the single-sensor HARP, GC 00, was calculated based on the estimated position of the tracked whale at each second. The off-axis angle is informative because beaked whale echolocation clicks are highly directional, therefore, the detection range depends on the animal’s orientation relative to the sensor. A generalized additive model (GAM) with a binomial response and logistic link function was used to model the probability of detecting beaked whale clicks as a function of range and off-axis angle to the sensor (GC 00). GAMs offer flexibility by avoiding the need to specify a parametric form for the function, instead utilizing smooth yet adaptable spline functions [55]. The model does not assume a detection probability of one at zero range, nor does it enforce a monotonic decrease in detection probability with increasing range. Fitting is via maximum likelihood with the amount of smoothness of the splines being selected as part of the maximization. Because the covariate values (distance and off-axis angle) were known, the detection probability can be predicted conditional on these values and an average taken. To derive the detection probability as a function of the distance alone, the off-axis angle was “integrated out” by assuming a uniform distribution of headings between 0° and 180° and averaging the detection probabilities for all angles at a given distance. The model was implemented using the library *mgcv* in R version 4.4.0 [56].

## Results

Over the 200 days in the Sep 7, 2021 to Mar 25, 2022 recording period for the two tracking HARPs, goose-beaked whale clicks were detected in 29 acoustic events, Gervais’ beaked whale clicks were detected in 54 acoustic events and Blainville’s beaked whale clicks were detected in two acoustic events (S2 Fig). After removing events for which both HARPs did not each include at least 100 clicks, 16 goose-beaked whale acoustic events, 20 Gervais’ beaked whale acoustic events, and the two Blainville’s beaked whale acoustic events were selected for further analysis.

### Localization and tracking

**Fig 3:**
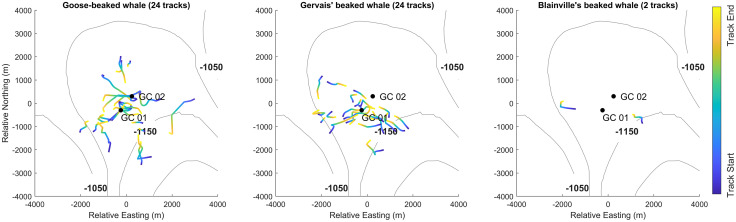
2D tracks (colored lines) and HARP locations (black dots) for all acoustically tracked dives for each of the three beaked whale species: goose-beaked (left), Gervais’ (middle), Blainville’s (right) beaked whales. Track colors indicate whales’ travel directions (start location blue, end location yellow). Tracking HARPs (GC 01, GC 02) indicated by black dots. Bathymetry is indicated by the 1050–1200 m isobaths, in 50m increments (gray lines). Easting and Northing are relative to the array center.

Of the analyzed acoustic events, the most commonly encountered minimum group size for goose-beaked and Blainville’s beaked whales were single animals (8 of 16 events); whereas, Gervais’ beaked whales were mostly encountered as groups at minimum of two animals (10 of 20 events) (S3 Fig). In several events, tracks of individuals were sufficiently distinct to be tracked separately (S1 Fig). Some tracks were rejected because they did not have unambiguous localizations or were too short (less than 7 min). In total, 24 goose-beaked whale tracks from 16 events, 24 Gervais’ beaked whale tracks from 20 events, and 2 Blainville’s beaked whale tracks from 2 events were used for dive parameter estimation (Fig 3).

**Fig 4:**
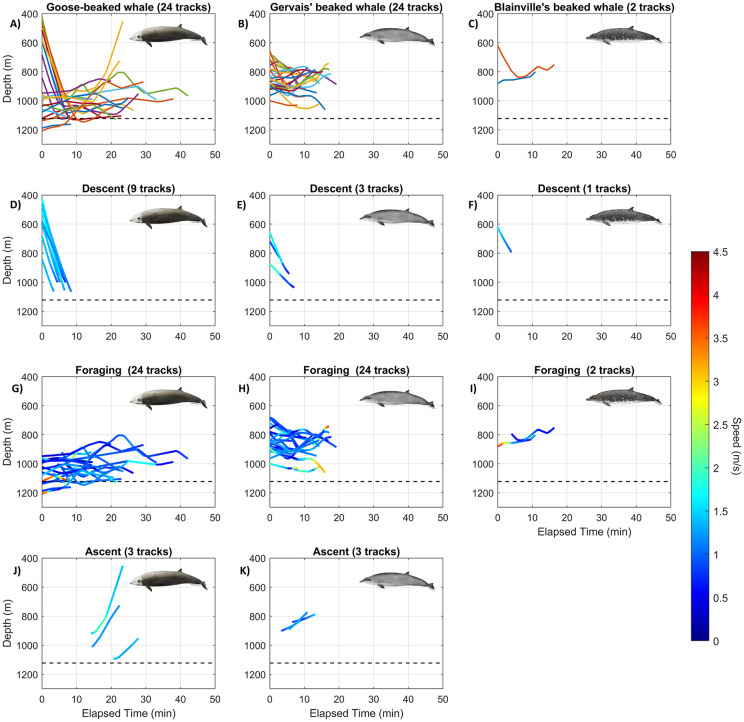
Elapsed time (minutes) and estimated depths (m) of tracked beaked whale dives for goose-beaked (A; 24 tracks), Gervais’ beaked (B; 24 tracks) and Blainville’s beaked (C; 2 tracks) whales. **(D–F)** Swim speed (m/s) for descent tracks for each of the 3 species. **(G–I)** Swim speed (m/s) for foraging (bottom) tracks for each of the 3 species. Swim speed (m/s) for ascent tracks for goose-beaked **(J)** and Gervais’ beaked **(K)** whales. Dashed black line indicates seafloor mean depth between two tracking HARPs.

Goose-beaked whale clicks from tracks were detected at a maximum range of 3.3 km (Fig 3, S1 Table) at almost all bearings around the GC sites, but clicks were mainly absent from the quadrant to the northeast of GC 02 where, seafloor depths were shallower (Figs 3 and S4). Gervais’ beaked whale clicks from tracks were detected at a maximum range of 2.7 km (Fig 3, S2 Table) and occurred across all bearings around the GC sites, with peaks to the east and west of GC 01 (Figs 3 and S4). There were only two Blainville’s beaked whale tracks, one to the southeast and one to the west of the array (Figs 3 and S4) and clicks from these tracks were detected at a maximum range of 2.4 km (Fig 3, S3 Table). The durations of acoustically-tracked goose-beaked whale dive segments were significantly longer (Tukey–Kramer’s HSD, p < 0.001) on average (mean: 20.5 min, range: 7–42 min) than those of Gervais’ (12.7 min; 7–19 min) and Blainville’s beaked whales (13.6 min; 11–16 min; Fig 4, Table 2).

### Dive behavior parameter estimates

The mean depth of goose-beaked whales was 983 m (sd = 95 m), which was significantly deeper (Tukey–Kramer’s HSD, p < 0.01) than the mean depths of 865 m (sd = 71 m) for Gervais’ beaked whales and 795 m (sd = 36 m) for Blainville’s beaked whales (Fig 4, Table 2). Similarly, the mean maximum dive depth of 1078 m (sd = 71 m) was significantly deeper for goose-beaked whales (Tukey–Kramer’s HSD, p < 0.001) compared to the mean maximum dive depths of 910 m (sd = 71 m) for Gervais’ beaked whales and 861 m (sd = 21 m) for Blainville’s beaked whales (Fig 4, Table 2). The dive altitude was estimated using the average seafloor depth (~1100 m) and the mean dive depth. In the Monte Carlo simulation, since goose-beaked whales spent a significant portion of their dive near the seafloor (see Fig 4), their dive altitude mean was restricted to 100–110 m, with a standard deviation of 50–60 m (cf. Table 3). In contrast, the dive altitudes for Gervais’ and Blainville’s beaked whales, which exhibited less consistent diving behavior, were rounded to the nearest hundred: Gervais’ beaked whale at 200–300 m (standard deviation: 50–100 m) and Blainville’s beaked whale at 300–400 m (standard deviation: 50–100 m).

Nine descent tracks of goose-beaked whales were observed, with starting depths ranging from 425 to 842 m. Interestingly, in goose-beaked whales, 7 out of 9 detected descent tracks began between 425 and 597 m. Only three ascent tracks of goose-beaked whales were observed, with finishing depths varying considerably, ranging from 452 to 952 m. For Gervais’ beaked whales, descent tracks were observed starting at depths of 657–874 m (n = 3), while ascent tracks concluded at depths of 772–815 m (n = 3). The only detected descent track for Blainville’s beaked whale started at 621 m. All parameters derived from our study were used where we were confident in their estimates. For the Monte Carlo simulation, we used an average depth at start of clicking of 450–460 m for goose-beaked whales and literature-based values of 420–430 m for Blainville’s beaked whales [Blainville’s beaked whales, 4]. Because the depth at the start of clicking for Gervais’ beaked whales remains uncertain, as only a limited portion of the descent phase was available for this species, we conducted multiple Monte Carlo simulations using different values for this parameter. These analyses indicate that using Blainville’s beaked whale data provides a suitable approximation for Gervais’ beaked whales, as *in situ* measurements show good agreement with the cue-counting model’s predicted distributions of horizontal range, elevation angle, and received level.

Foraging track swim speeds were similar (Tukey–Kramer’s HSD, p = 0.28) among the three species (Fig 4, Table 2). The overall mean swim speeds of goose-beaked, Gervais’ beaked and Blainville’s beaked whales were 0.99 m/s (sd = 0.32 m/s), 1.13 m/s (sd = 0.28 m/s), and 1.09 m/s (sd = 0.36 m/s), respectively. Mean descent rate for Gervais’ beaked whales was 1.15 m/s (sd = 0.16 m/s, *n* = 3), and was similar to the mean descent rates of 1.14 m/s (*n* = 1) for Blainville’s beaked whales and 1.34 m/s (sd = 0.15 m/s, *n* = 9) for goose-beaked whales (Fig 4, Table 2). Mean ascent rate of 1.40 m/s (sd = 0.16 m/s, *n* = 3) for goose-beaked whales was similar (Tukey–Kramer’s HSD, p = 0.1) to the ascent rate of 1.19 m/s (sd = 0.06 m/s, *n* = 3) for Gervais’ beaked whales (Fig 4, Table 2). Neither of the Blainville’s beaked whale tracks presented an ascent; thus, the ascent rate was not estimated for this species.

The average pitch angle in the descent was steeper than in the ascent for both goose-beaked and Gervais’ beaked whales (Tukey–Kramer’s HSD, p < 0.001). The descent pitch angle of goose-beaked whale averaged 68.5° (sd = 8.83°), which was steeper than that of both Gervais’ (35.7°, sd = 8.51°) and Blainville’s (36.3°) beaked whale (Tukey–Kramer’s HSD, p < 0.001). The ascent pitch angle in goose-beaked whales averaged 26.1° (sd = 8.95°), which was not statistically different (Tukey–Kramer’s HSD, p = 0.07) from the 12.1° (sd = 4.68°) pitch ascent observed in Gervais’ beaked whales (Table 2). For the Monte Carlo simulation, we used an average descent pitch angle of 70–75° for goose-beaked whales, 35–40° for Gervais’ beaked whales, and literature-based values of 72–77° (74° average from Tyack et al. 2006) for Blainville’s beaked whales, as our sample size (n = 1) was too small to be reliable (cf. Table 3).

### Acoustic behavior parameter estimates

**Fig 5:**
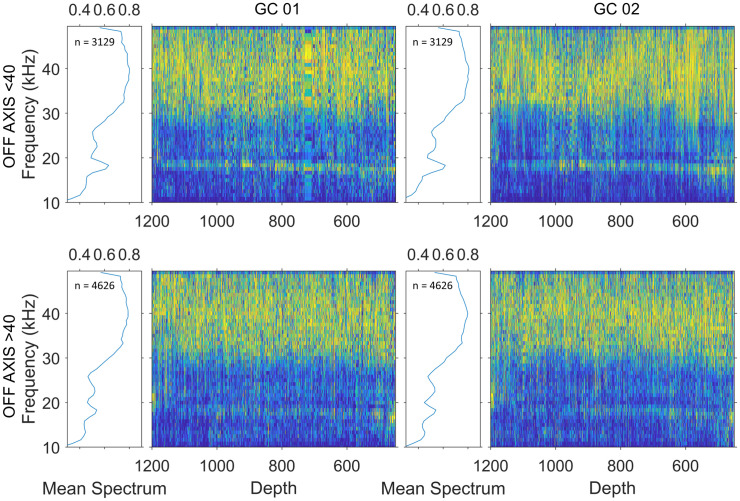
Mean normalized spectrum and spectrogram of concatenated normalized click spectra sorted by depth for localized and tracked goose-beaked whale clicks at GC 01 (left set of panels) and GC 02 (right set of panels) when off-axis angles are less than 40° (upper set of panels) or greater than 40° (lower set of panels).

For the click detections associated with high-quality tracks, the peak frequency for Gervais’ beaked whale clicks (mean: 42.7 kHz ± 4.52 kHz) was higher than the peak frequency for goose-beaked whale clicks (39.3 kHz ± 6.29 kHz) and the peak frequency for Blainville’s beaked whale clicks (35.3 kHz ± 4.75 kHz) (Tukey–Kramer’s HSD, p < 0.0001). Interestingly, while peak frequency remained the same, minor spectral peaks (at 17 and 23 kHz) in the goose-beaked whale clicks seemed to increase in frequency as whale depth increased, suggesting possible influence of pressure on spectral features, and disappeared at large off-axis angles, suggesting different beam-related propagation loss at low frequencies (Figs 5 and S5). No changes in the overall shape of the spectrum or shift in the peak frequency with depth or off-axis angles were observed in Gervais’ and Blainville’s beaked whale clicks (S5 Fig). For the Monte-Carlo simulation (cf. Table 3), echolocation click peak frequency was rounded to the nearest even number (Goose-beaked whale and Gervais’ beaked whale: 40 kHz; Blainville’s beaked whale: 34 kHz).

The ICI of echolocation clicks was estimated by removing ICIs less than 0.1 s and greater than 1 s to avoid including foraging buzzes and pauses [38]. Goose-beaked whales had the highest modal ICI, at 535 ms, followed by Blainville’s beaked whales at 304 ms and Gervais’ beaked whales at 276 ms. In goose-beaked whales, during the descent phase, the ICI was reduced from 0.9 to ~0.4 s (S6 Fig). No adjustment of the ICI was observed during the descent phases in Gervais’ or Blainville’s beaked whales.

**Fig 6:**
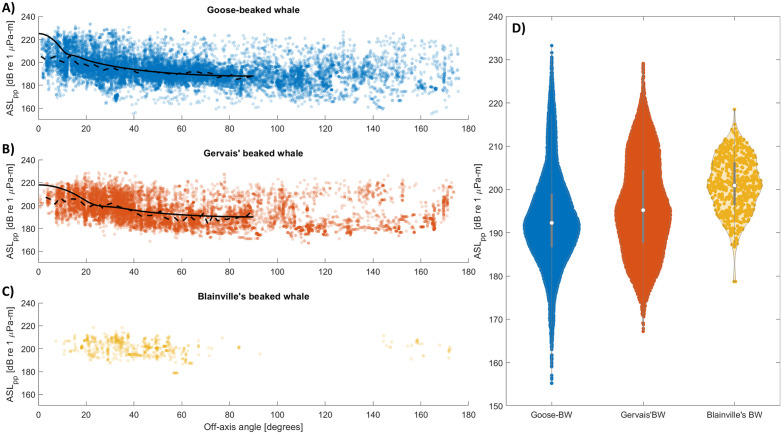
A–C: Peak to peak apparent source level estimates (ASL_pp_) of goose-beaked (n = 15,728 clicks), Gervais’s beaked (n = 12,856 clicks), and Blainville’s beaked (n = 676 clicks) whale clicks from the tracks as a function of off-axis angle to GC 01 and GC 02. Median ASL_pp_ for each off-axis angle bin (bin size 2°; dashed line) and modeled beam pattern attenuation predicted for a piston (solid line) with a broadband DI equivalent to 26 dB and an ASL of 225 dB re 1 µPa-m for goose-beaked whale and a broadband DI equivalent to 20 dB and an ASL of 218 dB re 1 µPa-m for Gervais’ beaked whale. D: Violin plots of the apparent source level distribution for each species. Violin plots are used to show the probability density of the data at different values. The white dot represents the median, the thick gray bars inside each violin plot represent the interquartile range, and whiskers are the minima and maxima.

A variability pattern in received levels was observed in a tracked goose-beaked whale (S7 Fig) with peaks of over 20 dB difference on timescales of approximately 1 minute, suggesting scanning movements of the animal’s head coupled with the beam pattern of clicks. To quantify the directionality of the clicks produced by Gulf beaked whales, the ASL_pp_ estimates of tracked clicks recorded on GC 01 or GC 02 were evaluated as a function of their angle away from the whale’s long axis of orientation (i.e., off-axis angles; Fig 6). For both goose-beaked and Gervais’ beaked whales, the median ASL_pp_ decreased from on-axis (0°) to 90° off-axis, but this was less evident for Blainville’s due to limited data. Across the three species, very few clicks were recorded at off-axis angles <10°, particularly for Gervais’ and Blainville’s beaked whales. The majority of strong ASL_pp_ values fell between 15–30° for goose-beaked whales and between 20–40° for Gervais’ and Blainville’s beaked whales. For goose-beaked, Gervais’ and Blainville’s beaked whales, the highest ASLs_pp_ at any angles were 233, 229 and 218 dB_pp_ re 1 µPa-m, respectively. A radial broadband piston beam pattern fitted to the median ASL_pp_ distribution over the 0–90° angle range yielded median SL_pp_ estimates of 225 and 218 dB re 1 µPa-m for goose-beaked and Gervais’ beaked whales respectively, and DI of 26 dB and 20 dB for goose-beaked and Gervais’ beaked whales respectively. SL_pp_ and DI could not be estimated for Blainville’s beaked whales due to the limited data. For the Monte Carlo simulation, from our observations, we used an average source level of 223–228 dB_pp_ re 1 µPa-m for goose-beaked whale, 215–220 dB_pp_ re 1 µPa-m for Gervais’ beaked whale, and literature-based values of 215–220 dB_pp_ re 1 µPa-m [57] for Blainville’s beaked whale (cf. Table 3). For the Monte Carlo simulation, from our observations, we used a DI of 24–28 dB for goose-beaked whale, and 18–22 dB for Gervais’ beaked whale, and literature-based values of 21–25 dB [57] for Blainville’s beaked whale (cf. Table 3).

### Detection probability

**Fig 7:**
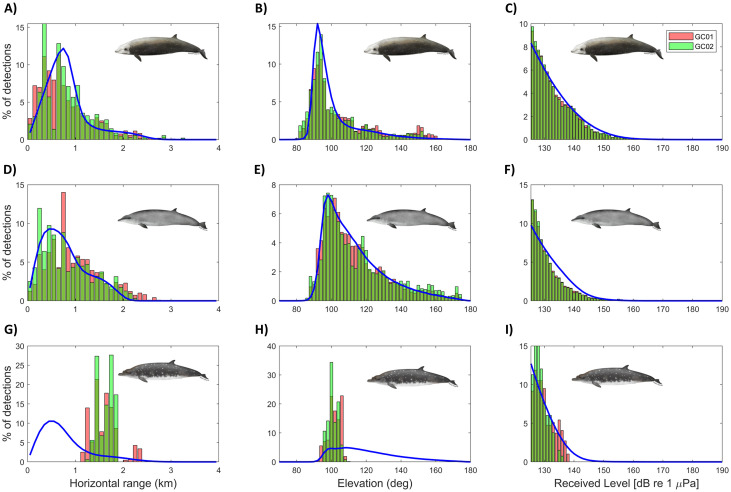
Comparison of *in situ* horizontal ranges, elevation angles and received levels of detected clicks (overlaid; GC 01: red bars and GC 02: green bars) with cue-counting model predicted distributions for each variable (blue lines). Solid blue lines indicate model-predicted mean across 500 model iterations. Top **[A – C]**: goose-beaked whale encounters. Center **[D – F]**: Gervais’ beaked whale encounters. Bottom: **[G – I]**: Blainville’s beaked whale encounters. Columns of plots from left to right show horizontal range (*in situ*: tracked beaked whales), elevation (*in situ*: all detected beaked whales) and received level (*in situ*: all detected beaked whales) distributions. For easier viewing, these plots are on different Y-axis.

The probability of detecting beaked whale clicks as a function of horizontal range was modeled using a simulation method that incorporated parameters from this study and/or literature. Parameter values were considered a good fit when model predictions aligned with *in situ* data. For example, we initially used 28–32 dB_pp_ (based on the goose-beaked whale study by Zimmer et al. [43]) for both side and back minimum off-axis amplitude loss across all three species. However, we found that 38–42 dB_pp_ for the back minimum off-axis amplitude loss of Gervais’ and Blainville’s beaked whales provided a better fit, as model predictions more accurately matched *in situ* observations. Cue-counting model predictions (individual level) using Monte Carlo simulations estimated that an average of 6.8% (sd = 1.0) of goose-beaked, 3.6% (sd = 0.7) of Gervais’ and 3.2% (sd = 0.8) of Blainville’s beaked whale clicks produced within 4 km of the sensor would be detected (S4 Table). For all 3 species, the probability of detection decreased with range (Fig 7). The simulation estimated a maximum goose-beaked whale click detection range of 3.6 km, which is slightly larger than the observed maximum localization distance of 3.3 km (Fig 7, S1 Table). On the contrary, the simulation estimated a maximum Gervais’ beaked whale click detection range of 2.5 km, which is slightly smaller than the observed maximum localization distance of 2.7 km (Fig 7, S2 Table). Horizontal range, elevation angle and received level distributions were similar between the model predictions and *in situ* values for both goose-beaked and Gervais’ beaked whales (Fig 7). Both horizontal range and elevation angle distribution for Blainville’s clicks differed between the model predictions and *in situ* values due to the small sample size (Fig 7).

**Fig 8:**
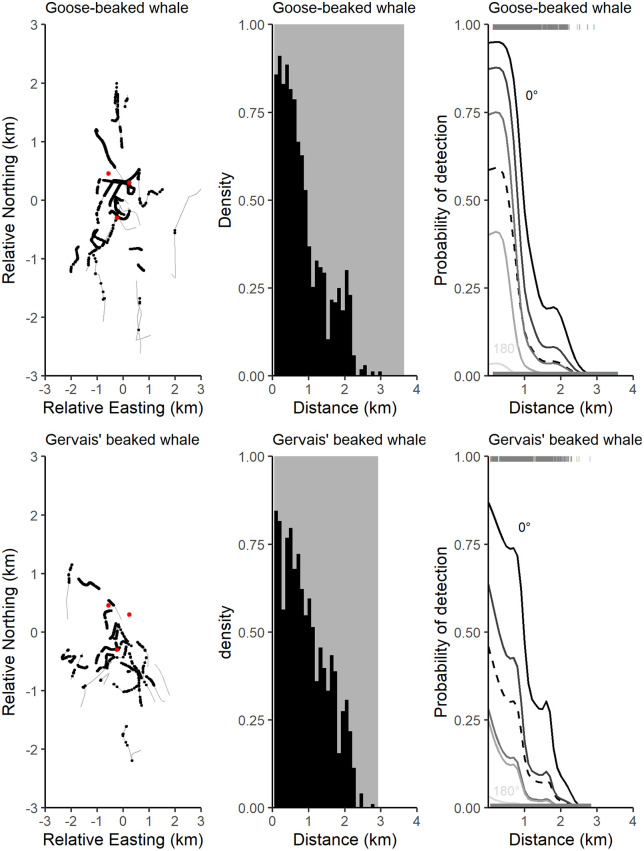
Trial-based approach results. Left) Acoustically tracked beaked whale tracks (grey) including positions where clicks were detected (black) on GC 00. The two panels represent each of the two species: goose-beaked (top), and Gervais’ (bottom) beaked whales. Single-channel HARP (GC 00) and Tracking HARPs (GC 01, GC 02) indicated by red dots. Middle) Proportion of detected (black) and not-detected (grey) beaked whale clicks as a function of horizontal ranges (km). Right) Trial-based estimates of detection probabilities for beaked whale clicks are presented as a function of horizontal range (in km), derived from a binomial GAM fit to detection and non-detection data. Solid lines represent the detection probabilities for on-axis clicks (0°, black) and off-axis clicks (45°, 90°, 135°, and 180°, shown from dark to light gray). The dashed line represents the detection probability averaged over all off-axis angles. Detected (1) and not detected (0) clicks shown as grey lines at the top and bottom of the plots. Detection probabilities for Blainville’s beaked whales were not estimated due to insufficient sample size.

**Fig 9:**
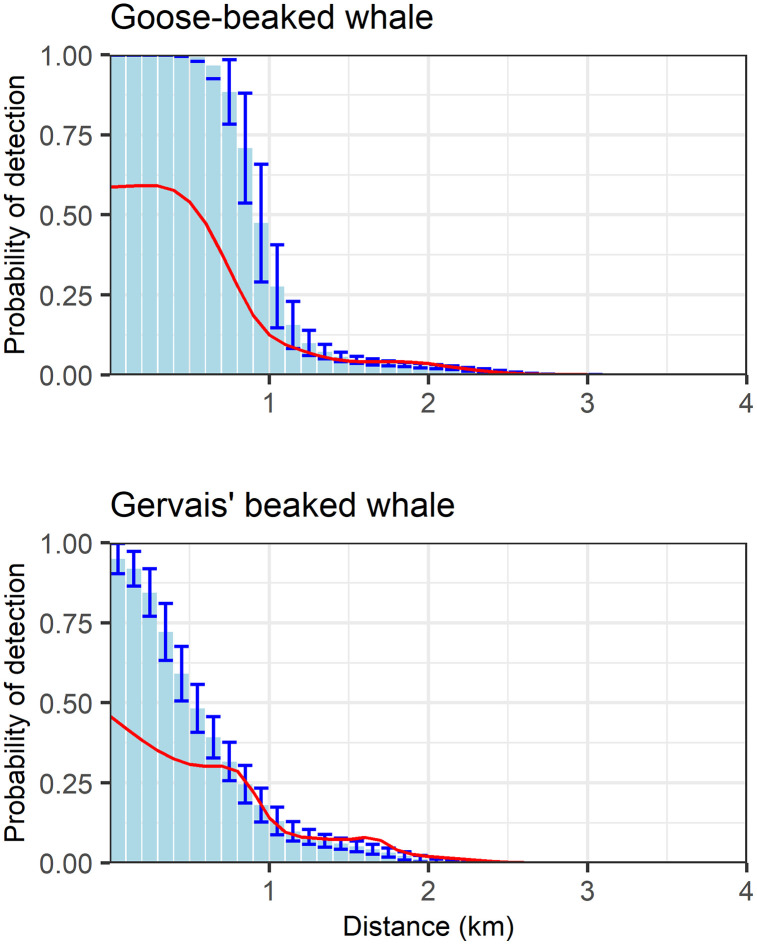
Comparison of estimated detection probabilities for goose-beaked (Top) and Gervais’ (Bottom) beaked whale clicks as a function of horizontal range (in km) based on the binomial GAM with the detection probability averaged over all off-axis angles (red line) and based on the Monte-Carlo simulation (blue bars, show + /- one standard deviation).

Results from the trial-based binomial fitting of tracked goose-beaked and Gervais’ beaked whale click detections on the independent third HARP (Fig 8) indicated detection probability generally declined with increasing range and increasing off-axis angles, although the decrease with the range was not monotonic. When comparing probability of detection between cue-based detection probability simulation and trial-based estimation, differences in slope are observed at short horizontal distances (Fig 9). This is likely due to the low number of tracked beaked whale dives in the near vicinity of GC 00. However, detectability showed greater similarity at longer horizontal distances. The detection probability fell sharply for greater horizontal ranges, owing to the highly directional beam pattern of beaked whale click production.

## Discussion

Input parameters used for population density estimation should ideally be spatially relevant and population-specific. This study provides the first detailed descriptions of echolocation click parameters and *in situ* diving behavior for three species of beaked whales in the Gulf, where diving and acoustic behavior data were previously lacking. This information is needed to improve the accuracy of detection probability simulation models for estimating density using distance-sampling point-transect methods for single-sensor-based passive acoustic monitoring. Key features of bioacoustic signals that may influence the probability of detection of a signal include source level and directionality. The passive acoustic tracking methods applied in this study provide a non-invasive tool to study acoustic parameters and subsurface dive behavior of these elusive, cryptic species, which are challenging to tag to obtain these data directly. The results of this study augment what is known from beaked whale diving and acoustic behavior tagging studies by tracking a larger number of individuals over a longer time period, with 50 tracks obtained over the 6 months recording period, and provide the first such information for the Gulf.

### Temporal and spectral characteristics

Deepening our understanding of beaked whale acoustic behavior, including variations in cue production rates, improves our ability to accurately assess their populations using passive acoustics. Cue rate is a critical multiplier in density estimation methods based on cue counting methods, and incorrect multipliers result in incorrect estimates of density. The dive cycle click production rates (cue rates) for beaked whales are estimated by taking the mean proportion of a dive cycle spent clicking and multiplying by the inverse of the average ICI [14]. Sperm whales and pilot whales have been observed adjusting their ICI during deep dive descents to track the seafloor or a prey layer [58–60]. In our study, goose-beaked whales showed a similar downward ICI adjustment, which has also been reported by Gassmann et al. [26], suggesting that they may be tracking the seafloor as they forage along it. However, no similar adjustments were found in our study for Gervais’ or Blainville’s beaked whales, which forage more pelagically [1,31,61]. Goose-beaked whale ICIs (mode: 535 ms; echolocation clicks) measured in this study are longer than those reported in other regions [ICI: 337–465 ms; 7,41–44] but similar to values previously found in the Gulf [ICI: 500–544 ms; 14,62]. Hildebrand et al. [14] reported some spatial variation among sites in the Gulf, with slightly longer modal ICIs for goose-beaked whales at the DT site (530 ms) relative to the GC site (520 ms) and the MC site (500 ms). Modal ICIs in this study for Gervais’ (276 ms) and Blainville’s beaked whales (304 ms) were similar to other studies in the Gulf and elsewhere; Gervais’ beaked whale [ICIs: 270–290 ms; 7,14,19,62], Blainville’s beaked whale [ICIs: 290 ms – 400 ms; 14,38,39,63]. Recently, Baumann-Pickering et al. [63] demonstrated that ICIs of Blainville’s beaked whale echolocation clicks varied geographically, which emphasizes the importance of using population-specific parameters.

In the Monte Carlo simulations, received levels at the sensor are calculated for clicks produced at each modeled source position, using the assigned source parameters and model-based transmission loss estimates at the click peak frequency. Thus, for modeling purposes, the mean peak frequency of received clicks is a critical value. Although Gervais’ beaked whale signals are similar to those of goose-beaked and Blainville’s beaked whale signals, with energy concentrated in the 20–50 kHz band, they have a slightly higher peak frequency than those of the other two species [19]. Mean values of 39 kHz, 43 kHz, and 35 kHz, found in this study for goose-beaked, Gervais’, and Blainville’s beaked whales, respectively, were in good agreement with previously published estimates of 40 kHz [goose-beaked whales, 7,43], 40–44 kHz [Gervais’ beaked whales, 7,16] and 32–40 kHz [~34 kHz in the Gulf, Blainville’s beaked whales, 63]. It is important to note that the tracking HARP sample rate (100 kHz) is low for understanding the full acoustic properties of Gervais’ beaked whale clicks with significant amounts of energy expected above the 50 kHz Nyquist cutoff frequency [19]. The peak frequency of Blainville’s beaked whale echolocation clicks varies geographically, with lower peak frequencies in higher latitudes [63], highlighting the importance of using population-specific parameters to estimate peak-frequency for use in estimating frequency-dependent transmission loss.

The Monte Carlo simulation relies on the assumption that species-specific signal characteristics will be consistent across depth. Interestingly, for goose-beaked whale clicks, the minor spectral peaks increase in frequency as depth increases, and disappear at large off-axis angles; however, the peak frequency was not affected. One hypothesis to explain that is that, in goose-beaked whales, air present between the phonic lips [the structural complex that produces echolocation clicks, 64] produces smaller bubbles when pressurized, which modify frequency content at lower frequencies. Our observation highlights the need to investigate whether hydrostatic pressure (depth) or off-axis effects affect the click characteristics of parameters included in simulations, to ensure that variation is accounted for.

### Foraging time and dive depths

Beaked whale tagging studies show that deep dives are primarily for foraging [39,42], and the duration of acoustic activity (echolocation clicks) reflects the length of foraging bouts. Near Green Canyon, goose-beaked whale individuals were acoustically detected for longer periods (mean: 20.5 min; range: 7–42 min) than Blainville’s (13.6 min; range: 11–16 min) or Gervais’ (12.7 min; range: 7–19 min) beaked whales. However, these acoustic activity periods likely represent only a portion of their actual foraging time, given the narrow beam angle of echolocation clicks and frequent changes in dive direction [24,26]. Previous studies, using animal-mounted acoustic recorders reported that Ligurian Sea (Mediterranean) goose-beaked whales dive for ~ 60 min [34], with acoustic activity periods ~35 min observed there and in Southern California [21,34]. In contrast, Blainville’s beaked whales in the Canary Islands exhibit dives lasting ~50 min [34] with 24 and 30 min of acoustic activity recorded in the Canary Islands and the Bahamas, respectively [21,34]. While regional differences in acoustic activity period appear to exist for Blainville’s beaked whales, our lower estimate in the Gulf is likely the result of limited data. For Gervais’, only one full dive cycle (~ 88 min, period during which no whales were observed at the surface) has been documented with clicks detected for 15 min on a towed hydrophone array [19]. DeAngelis et al. [31] reported on towed array an acoustic event lasting ~32 min for Gervais’ beaked whales; however, because multiple animals were diving in close proximity, the duration attributable to a single individual could not be determined. The same authors reported the next longest detection duration for Gervais’ beaked whales as 15.4 min. Although little is known about this species, it may produce clicks for shorter periods than the other beaked whale species. Future efforts to tag Gervais’ beaked whales in the Gulf are needed to clarify acoustic and diving behavior of this species. The relationship between the complete dive cycle duration and the portion of the dive cycle spent acoustically active in beaked whales is crucial for density estimation using passive acoustics because if whales spend more time silent relative to their total dive cycle, the chance of detecting them in a given time window decreases, affecting density estimates.

In odontocetes, initial echolocation is presumably aimed at locating the patch or depth layer with the highest concentration of prey. In our study, 7 of the 9 detected descent dives for goose-beaked whales started between 425 and 597 m. Tracking studies using bottom-mounted hydrophones in Southern California [26] and studies using animal-mounted acoustic recorders in the Ligurian Sea [4,42] show that goose-beaked whales start echolocation during their descent at an average depth of 500 m (range: 136–531 m). Studies using animal-mounted acoustic recorders have shown that echolocation activities for Blainville’s beaked whales in the Canary Islands typically commence at an average depth of 400 m (range: 178–570 m) while the whales are descending [4,42]. Studies using animal-mounted acoustic recorders have also shown that echolocation activities typically ends at a depth of 856 m (goose-beaked whales; range: 588–1849 m) or 738 m (Blainville’s beaked whales; range: 500–860 m) while the whales are ascending [4,42]. In our study, detected ascents for goose-beaked whales finished around 710 m (sd: 200 m; range: 452–952 m, *n* = 3) with one individual echolocating up to 452 m depth. Echolocation clicks of the two other individuals were detected up to 952 and 725 m during their ascent dives. Values in our study for detected ascents for Gervais’ beaked whales (mean: 791 m; sd: 17 m; range: 772–815 m, *n* = 3) were similar to those reported in the literature for the two other beaked whale species. This suggests these ascents may represent the true ends of the acoustic activity periods for these dives.

Across all tracked dives, goose-beaked whales reached a mean value of maximum depth of 1078 m (888 m – 1208 m). This value is similar to the mean value of maximum depth for seven tagged individuals in deep (700–2000 m) waters of the Ligurian Sea [1070 m; 4], ten acoustically tracked (DASBRs) individuals in the Catalina Basin (nominal seafloor of 1250 m) off Southern California (1104 m; Barlow et al. [24]), and mean depth (1116 m ± 412 m) as detected on a towed array in the Western North Atlantic Ocean (mean seafloor ~ 2000 m) [31] but shallower than for other tagged goose-beaked whales in deep waters (~3000 m) of Southern California [8 individuals; 1401 m; 2], off Cape Hatteras, North Carolina [11 individuals; 1424 m; 20], or off Hawaii [2 individuals; 1392 m; 65]. This indicates that their diving behavior at GC site is constrained by the seafloor depth (~1100 m), with several animals foraging at or near the seafloor during at least a portion of their dive and animals being absent in the quadrant to the northeast of GC 02, where seafloor depths were shallower such that preferred prey may be unavailable at those depths (Figs 3 and S4).

In beaked whales, echolocation persists through an acoustically active bottom phase, indicating a sustained effort in identifying and pursuing potential prey during deep dives [4,39,42]. In this study, the mean foraging depth of Blainville’s beaked whales (795 m, sd = 36 m) was significantly shallower than that of goose-beaked whales (983 ± 95 m) and similar to that of Gervais’ beaked whales (865 ± 71 m). Moreover, the mean depth for clicks estimated with towed arrays off the U.S. Atlantic coast was between 783 m [sd: 399 m; 66] and 1158 m [sd = 287 m; 23] for goose-beaked whales, ~ 960 m for Blainville’s beaked whales [31], and between 661 m [sd = 309 m; 66] and 872 m [sd: 321 m; 31] for Gervais’ beaked whales. This study is the first to describe diving behavior for Gervais’ beaked whales. Our results suggest that this species display distinctly shorter acoustic activity phase and shallower dives than goose-beaked and Blainville’s beaked whales. The different depths used by these beaked whale species suggest different foraging behavior and possibly diet among beaked whale species. This is in agreement with stomach contents analyses showing that goose-beaked whales forage on much larger cephalopods than other beaked whale species [67]. Understanding the depths at which beaked whale species spend time can provide insights into their prey specialization, with these depths varying among prey species.

### Swimming speed and pitch

The results from this study extend previous findings on beaked whale swimming speed. The overall mean swim speed in the foraging (bottom) portion of the dive was similar for all three species. Foraging (bottom) speed for goose-beaked whales were consistent with those from previous tracking studies [0.64–3 m/s; 24,26]. In this study, descent and ascent rates were similar within each beaked whale species, but differed among beaked whale species, with descent and ascent rates (forward speed) of 1.34 and 1.40 m/s, respectively for goose-beaked whales, and 1.15 and 1.19 m/s, respectively, for Gervais’ beaked whales. Similarly, Martín López et al. [68] documented mean forward descent and ascent speeds of 1.3 and 1.3 m/s, respectively, for goose-beaked whales. The slower descent and ascent rates of Gervais’ beaked whales compared to goose-beaked whales suggest that the two species differ in their physiological capabilities.

Our cue counting simulation method relies on only two dive behavior parameters that affect the probability of detecting a click: animal depth (discussed earlier) and the pitch angle, which refers to the orientation of their body along the longitudinal axis relative to the horizontal plane. In this study, the average descent pitch angle of goose-beaked whales (69° ± 9°) was similar to a previous (tagged) study [72°, range: 60–83°; 4]; however, our ascent pitch angle (26° ± 9°; range: 14–36°) was lower than previously reported [35°, range: 13–58°; 4]. Descent pitch angles for both Gervais’ (36° ± 9°; range: 24–42°) and Blainville’s (36°) beaked whales were lower than goose-beaked whales and lower than expected for Blainville’s beaked whales when compared to previous tagged studies [74°, range: 62–82°; 4]. The moderate descent pitch angle for these two species in this study suggests that in our limited samples we detected only the final portion of the descents. It is important to note that in the previous study (tagged animals), the descent is considered to extend from the surface until the whale began to produce echolocation clicks; and likewise, the ascent was considered to start at the last echolocation click and end at the surface [4], which might explain some discrepancies on pitch angles between our study and previously published results. A possible explanation for the smaller sample size of ascent dives is that, during ascent, the primary orientation of their echolocation signals is directed upward and away from the tracking HARP, reducing the likelihood of detection by bottom-mounted hydrophones.

### Foraging strategies

Swim direction, and acoustic diel patterns are critical factors in detection and density estimation for beaked whales because they influence acoustic availability and detectability in passive acoustics. Swim directions from shallow to deeper waters along with observations of individuals foraging at or near the seafloor during part of their dives suggest that goose-beaked whales foraged with the gradient of the slope, a behavior also reported for Blainville’s beaked whales in the Canary Islands [1]. Gervais’ beaked whale swim directions, including horizontal and vertical changes, suggest that they might preferentially forage in the western-southern side of GC. In our study, beaked whale acoustic events were detected both during day and night (S2 Fig) and dive profiles were similar across these periods (S8 Fig) suggesting that beaked whales in the Gulf do not exhibit diel foraging depth patterns. Diel variation has been previously observed in tagged goose-beaked and Blainville’s beaked whales in Hawai‘i [65], off southern California [69] and off El Hierro, in the Canary Islands [1] with whales spending more time in silent, shallow dives (<100 m) at night than during the day. However, for deep foraging dives, the maximum dive depth, duration, and overall depth distribution of foraging effort change little between day and night [1,65], suggesting that whales primarily forage on non-migratory or partially migratory organisms of the deep scattering layer and on benthopelagic prey [1].

### Source level and directivity

One potential source of error in acoustic detection probability estimation from beaked whale echolocation clicks is the directional beam pattern effect on received levels on a sensor. Beaked whale clicks are highly directional [43], making them much more likely to be detected when the animal is directly oriented toward the sensor. In the cue counting method, the width of the beam pattern and the off-axis source level directly contribute to the overall detection probability. Specifically, the off-axis angle refers to the angle formed between the connecting line to the acoustic receiver and the clicking whale’s general direction of motion. A possible offset was observed in our ASL_pp_ distribution with a majority of strong ASL_pp_ values falling between 15–30° for goose-beaked whales and between 20–40° for Gervais’ and Blainville’s beaked whales. Similar offsets were also observed in previous studies of goose-beaked whales [18°–35°, 26,43]. This offset may result from the echolocation beam of the whale not being aligned with the rostral axis due to pronounced bilateral asymmetry [70]. Moreover, uncertainties in the off-axis angles might result from localization errors, errors from smoothing the curves, or errors in estimating the whale’s velocity vector and from its misalignment with the whales’ acoustic axis during clicking due to head and yaw movements [26]. We assumed that the average body axis aligns parallel to the mean direction of motion, implying that the body axis is generally parallel to the typical swim direction, but it might not always be true, which could explain why the decrease of the source level with the off-axis angle does not remain constant beyond 90° and why the “on-axis clicks” are not the strongest ones.

Accurate on-axis source level values ensure the simulation can realistically model how far beaked whales’ clicks can travel and remain detectable. In our study, the sparse distribution of ASLs (Fig 6) for beaked whales may reflect variations in the source level produced by the clicking whales [43]. The only published estimate of on-axis echolocation click amplitude for the goose-beaked whale was 214 dB_pp_ re 1 μ Pa-m, based on signals received from two simultaneously tagged animals in the Mediterranean Sea [43], however, this value is a lower bound due to clipping of the receiving array. In Gassmann et al. [26], apparent source levels up to 225 dB_pp_ re 1 µPa-m were estimated in acoustically tracked goose-beaked whales. Gassmann et al. [26] fitted a radial broadband piston beam pattern yielding a DI estimate of 30 dB, which is within the DI range previously reported for goose-beaked whale [> 25 dB; 43]. In our study, a radial broadband piston beam pattern was fitted to the median ASL_pp_ distribution of goose-beaked whale yielding a source level of 225 dB_pp_ re 1 µPa-m and a broadband DI of 26 dB, which are in agreement with those previously published values. For the Gervais’ beaked whale, no previous measurements of source level have been made. A source level of 218 dB_pp_ re 1 µPa-m and a broadband DI of 20 dB, similar to that reported for Blainville’s beaked whale [217 dB re 1 µPa-m and 23 dB, respectively; 57], appear to be reasonable estimates based on the observations in this study. We could not estimate source level for Blainville’s beaked whale due to our small sample size; however, in our study the highest ASL_pp_ was 218 dB_pp_ re 1 µPa-m, which is similar to the source level of 217 dB_pp_ re 1 µPa-m reported by Shaffer et al. [57].

### Detection probability

Density estimation using distance sampling methods requires accurately modeling the detection probability of the animals with respect to their horizontal distance from the acoustic sensor. The estimated detection function GAM for the trial-based case study of independent detections of tracked whales aligned with prior expectations: it decreased with increasing distance and off-axis angles, and high-source-level clicks from goose-beaked whales were detected at greater ranges than those of Gervais’ beaked whales. Detectability predictions are significantly influenced by click characteristics, notably source level, off-axis amplitude, and beam directivity [36]. For all three species, the majority of identified clicks were anticipated to be received off-axis, and the agreement between predicted (Monte-Carlo simulation) and *in situ* RLs and their distributions suggests that this assumption was true. Good agreement, notably for goose-beaked and Gervais’ beaked whale clicks, with *in situ* and predicted range and elevation angles suggests that Monte Carlo simulation provided a reliable estimate of click detectability. On the contrary, the discrepancy found in Blainville’s beaked whale clicks for both range and elevation angles can be explained by the small sample size for this species. Monte Carlo simulation models are designed to predict averages over many encounters; therefore, achieving perfect alignment with *in situ* data was not anticipated, especially considering the limited number of encounters (e.g., Blainville’s). Our results indicate that goose-beaked whales are most reliably detected (probability = 1) within 0.5 km, with a maximum detection range of 3.6 km. Similarly, Hildebrand et al. [14] reported the highest probability of detecting clicks only for horizontal ranges of less than 0.4 km and a maximum detection range of 3.5 km. In contrast, Li et al. [62] found higher values, with the highest probability of detecting clicks up to 1.4 km and a maximum detection range of 6 km. For Gervais’ beaked whales, we found the highest detection probability near 0.1 km and a maximum range of 2.5 km, slightly lower than Hildebrand et al. [14] (0.2 km, 3.2 km) and markedly lower than Li et al. [62] (0.7 km, 6 km). The differences are due to the species-specific parameters used (see S5 Table for a comparison). More precise input parameter estimates describing click characteristics and animal behavior would reduce uncertainty in the model predictions. Detection probability curves for both cue-counting model predictions (Monte Carlo simulations) and the trial-based approach (GAM) showed that probability of detecting goose-beaked whale clicks was higher than for Gervais’ beaked whale ones. This difference is primarily attributable to the greater proportion of time goose-beaked whales spend actively foraging, along with their higher source level and lower frequency clicks (Fig 9).

## Conclusion

Acoustic tracking is a reliable alternative to tagging for studying the acoustic and diving behavior of elusive beaked whales in the Gulf. While there are limitations to our methods and results, the data we present provides new information, notably regarding Gervais’ beaked whale diving habits and click parameters. Estimates of foraging time, dive depths, swimming speed, pitch angles, source level and directivity reported in our study are consistent with previous results from tagging and acoustic tracking studies in other areas and provide refined estimates of key variables needed to convert detection counts into density estimates using modeled detection probabilities. Follow-up studies to estimate whether values are consistent throughout the Gulf or are specific to the central-northern part of the Gulf are required. Finally, at this location, the foraging tracks of Gervais’ beaked whales were shorter and shallower than those of goose-beaked whales, indicating potential differences in foraging strategies and diet among beaked whale species. However, additional studies are required to confirm and elucidate these patterns.

## Supporting information

S1 Fig Example of three goose-beaked whale tracks (identified here as pink, blue and green) during one acoustic event. By observing gradual changes in both azimuth and elevation on both tracking HARPs (GC 01 and GC 02), it is possible to identify collections of detections originating from a single source. The azimuth is defined as the top-down counter-clockwise horizontal angle, where East is 0°, and North is 90°. The elevation angle is the vertical angle, where 0° is directly down, 90° is horizontal, and 180° is upward toward the sea surface.
(TIF)

S2 Fig Temporal occurrence of goose-beaked, Gervais’ beaked, and Blainville’s beaked whale clicks at the GC site. Blue dots represent any acoustic events with beaked whale clicks on GC 01 and/or GC 02. Red dots represent beaked whale detections selected for 3D tracking. Medium gray hourglass shading represents nighttime, while darker gray shading indicates periods of no effort.
(TIF)

S3 Fig Minimum group size distribution for goose-beaked, Gervais’ beaked and Blainville’s beaked whales derived from analyzed acoustic encounters at GC. The bars of each color add to 100%.
(TIF)

S4 Fig Histogram of measured bearing (in degrees) from the clicks produced by goose-beaked, Gervais’ beaked and Blainville’s beaked whales and detected at GC 02 (Top), GC 01 (Bottom) (all detections not only selected ones for tracking). In those plots, 0° indicates east and 90° indicates north. The length of the bar represents the number of click positive 1-min bins. Two tracking HARPs (GC 01 and GC 02) and bathymetry are displayed in the background.
(TIF)

S5 Fig Mean normalized spectra (A, D & G), spectrogram of concatenated normalized click spectra sorted by depth (B, E & H) and spectrogram of concatenated normalized click spectra sorted by off-axis angle (C, F & I) for goose-beaked whales (A–C), Gervais’ beaked whales (D–F) and Blainville’s beaked whales (G–I) at GC 01. (TIF)

S6 Fig Goose-beaked whale Inter-click intervals (ICI) as a function of depth during the 9 tracked descent phases (colored by track ID). (TIF)

S7 Fig Representative tracked dive for one goose-beaked whale (same event as Fig 2) at GC 01 recording location. Time series plot shows short-term variability in click received levels throughout the event that are typically indicative of orientation changes with scanning behavior.
(TIF)

S8 Fig Dive profiles during the day (top: red line) or at night (bottom: blue line) for goose-beaked whale (left) and Gervais’ (right) beaked whales. (TIF)

S1 Table Summary statistics of dive behavior and distance estimation parameters for each of twenty-four dive tracks for goose-beaked whales detected on the GC 01 and GC 02 tracking HARPs. (PDF)

S2 Table Summary statistics of dive behavior and distance estimation parameters for each of twenty-four dive tracks for Gervais’ beaked whales detected on the GC 01 and GC 02 tracking HARPs. (PDF)

S3 Table Summary statistics of dive behavior and distance estimation parameters for each of two dive tracks for Blainville’s beaked whales detected on the GC 01 and GC 02 tracking HARPs. (PDF)

S4 Table Mean predicted detection rates (%) and standard deviations obtained for each beaked whale species within a 4 km radius circular area around the HARP site. Estimates are based on 500 model iterations.
(PDF)

S5 Table Monte Carlo detectability simulation parameters by species and by study. (PDF)
