# Supplementary figures and images for: Beaked whale dive behavior and acoustic detection range off Louisiana using three-dimensional acoustic tracking
Source: PLoS One. 2026 Feb 4;21(2):e0340398. doi: 10.1371/journal.pone.0340398 (PMC12871975; doi:10.1371/journal.pone.0340398)

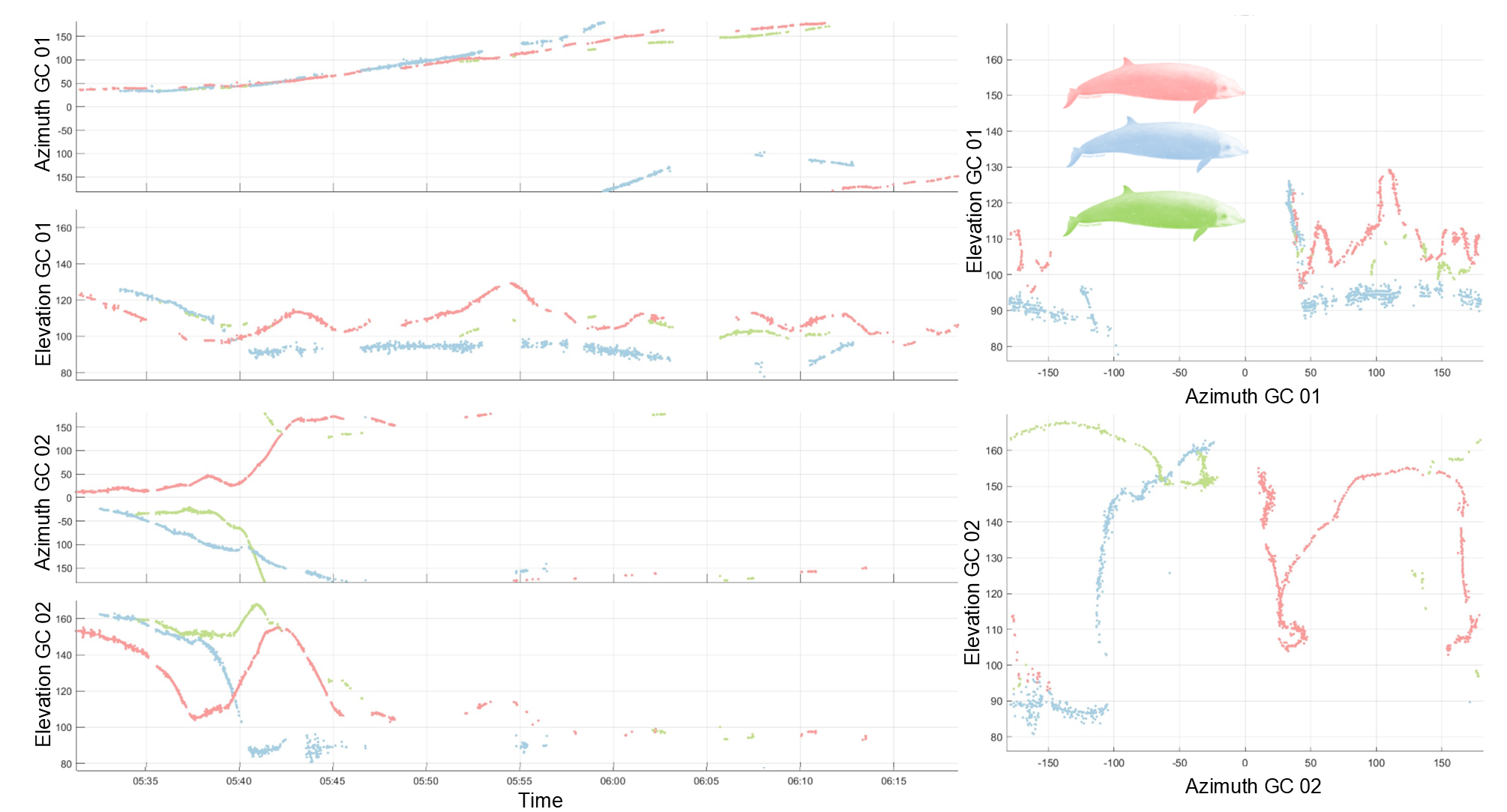

Supplement: S1 Fig — By observing gradual changes in both azimuth and elevation on both tracking HARPs (GC 01 and GC 02), it is possible to identify collections of detections originating from a single source. The azimuth is defined as the top-down counter-clockwise horizontal angle, where East is 0°, and North is 90°. The elevation angle is the vertical angle, where 0° is directly down, 90° is horizontal, and 180° is upward toward the sea surface. (TIF) [file pone.0340398.s001.tif]

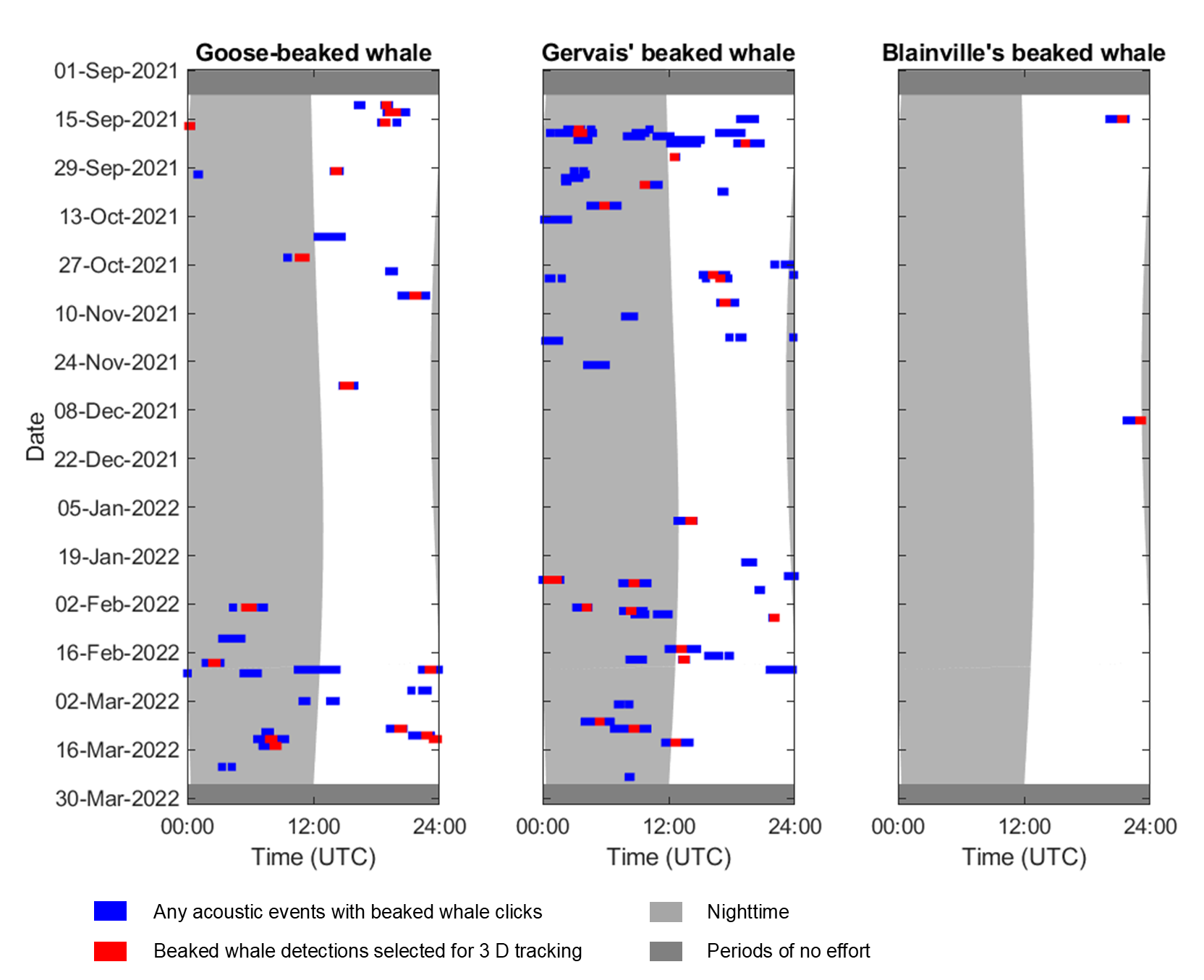

Supplement: S2 Fig — Blue dots represent any acoustic events with beaked whale clicks on GC 01 and/or GC 02. Red dots represent beaked whale detections selected for 3D tracking. Medium gray hourglass shading represents nighttime, while darker gray shading indicates periods of no effort. (TIF) [file pone.0340398.s002.tif]

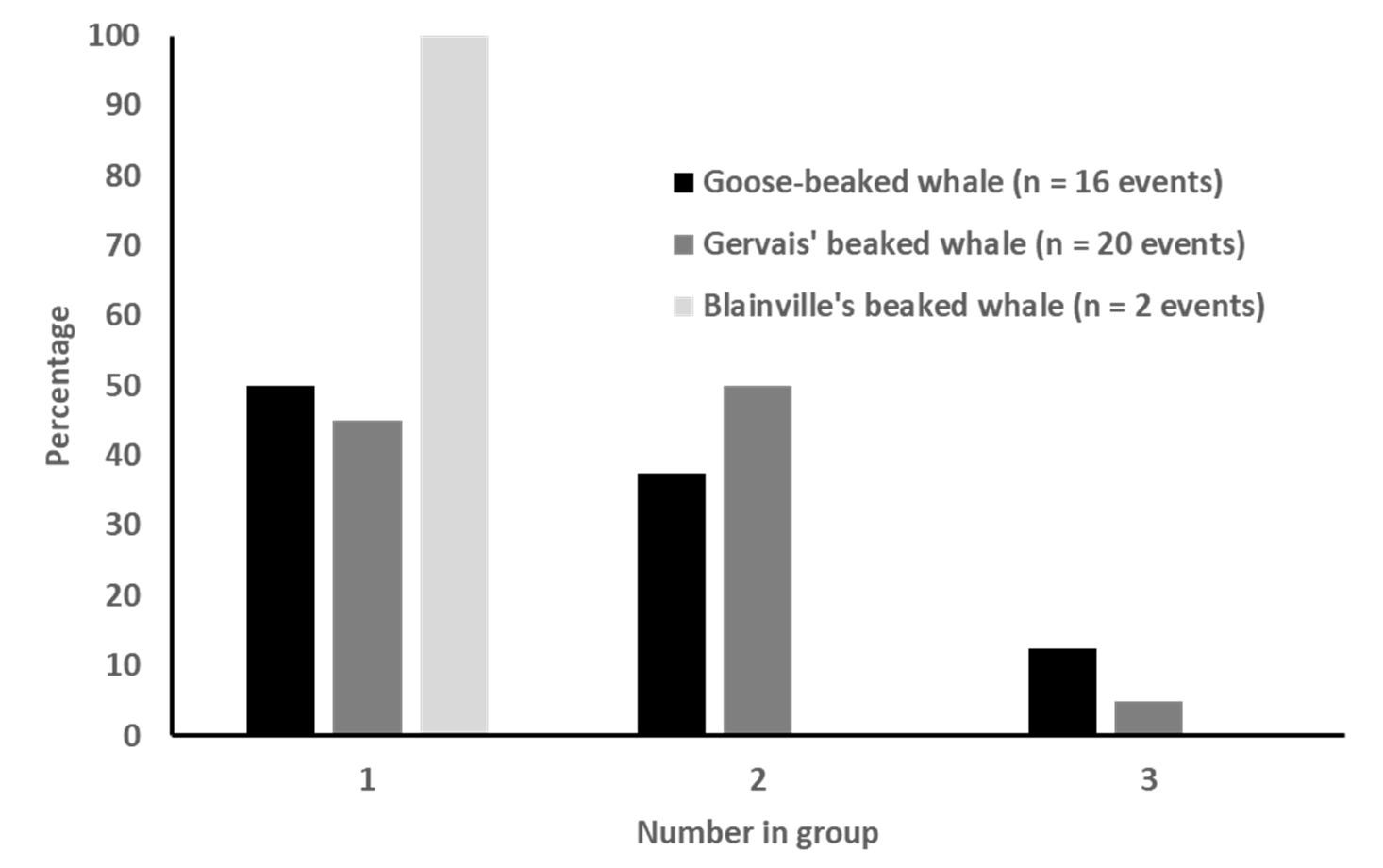

Supplement: S3 Fig — The bars of each color add to 100%. (TIF) [file pone.0340398.s003.tif]

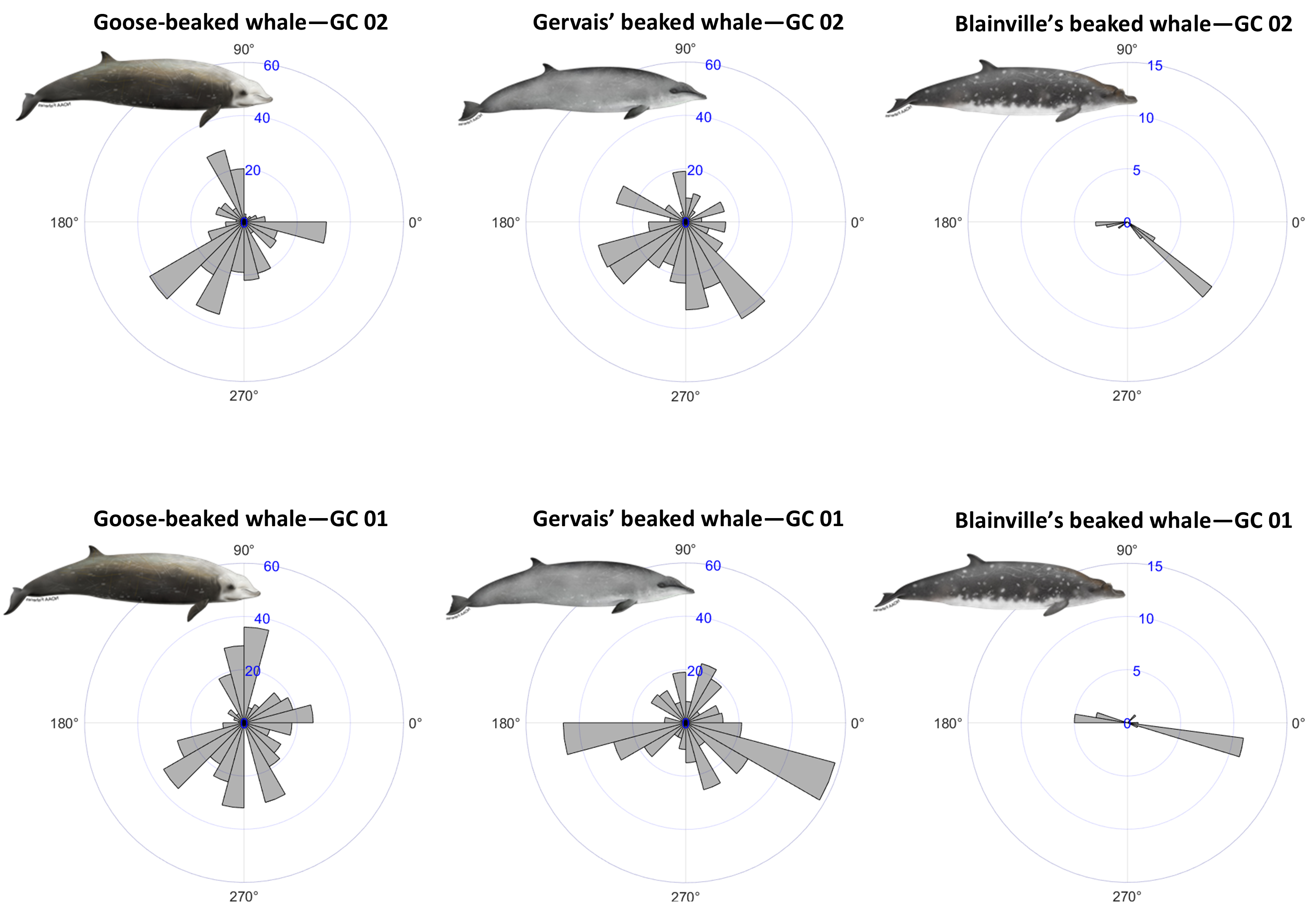

Supplement: S4 Fig — In those plots, 0° indicates east and 90° indicates north. The length of the bar represents the number of click positive 1-min bins. Two tracking HARPs (GC 01 and GC 02) and bathymetry are displayed in the background. (TIF) [file pone.0340398.s004.tif]

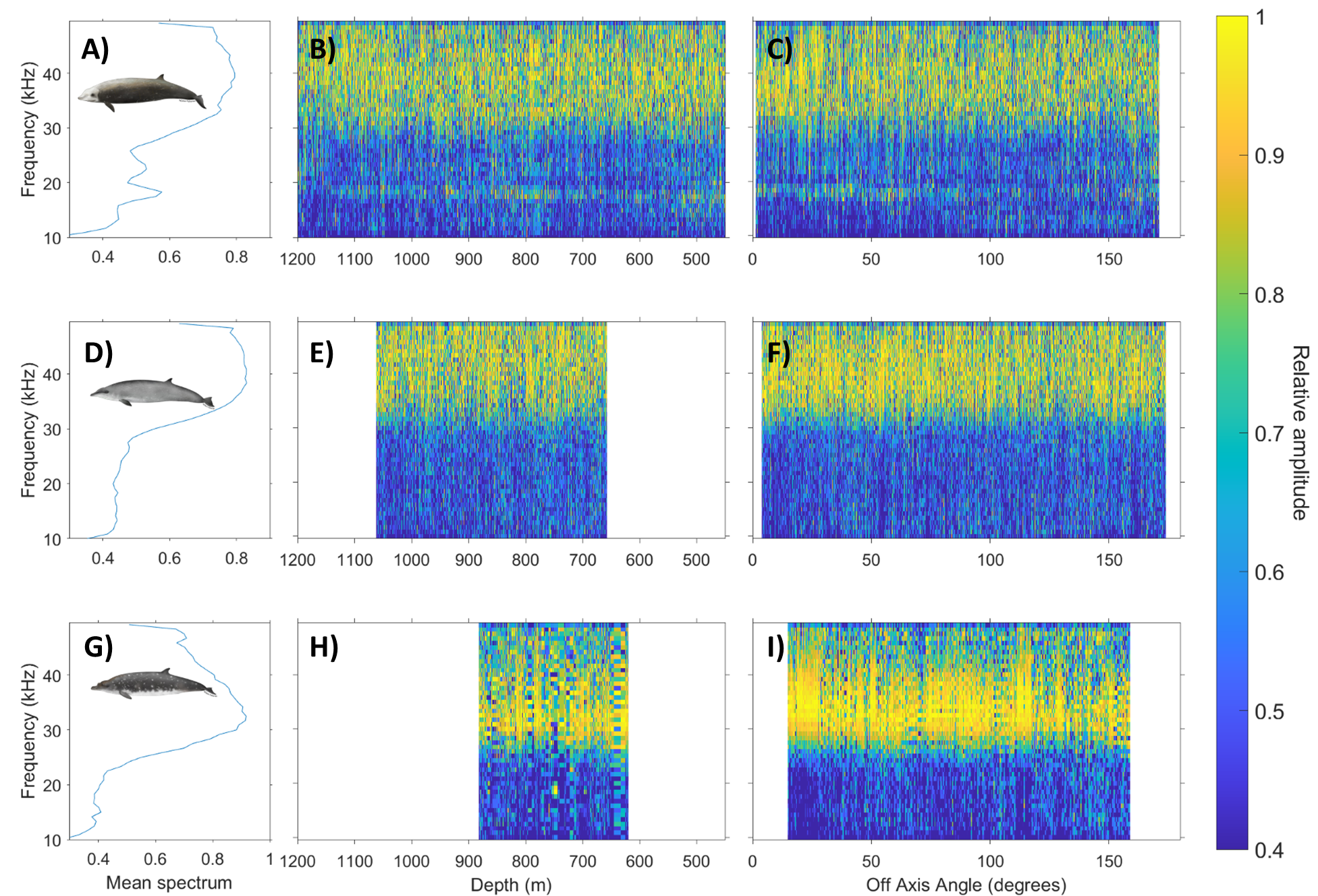

Supplement: S5 Fig — (TIF) [file pone.0340398.s005.tif]

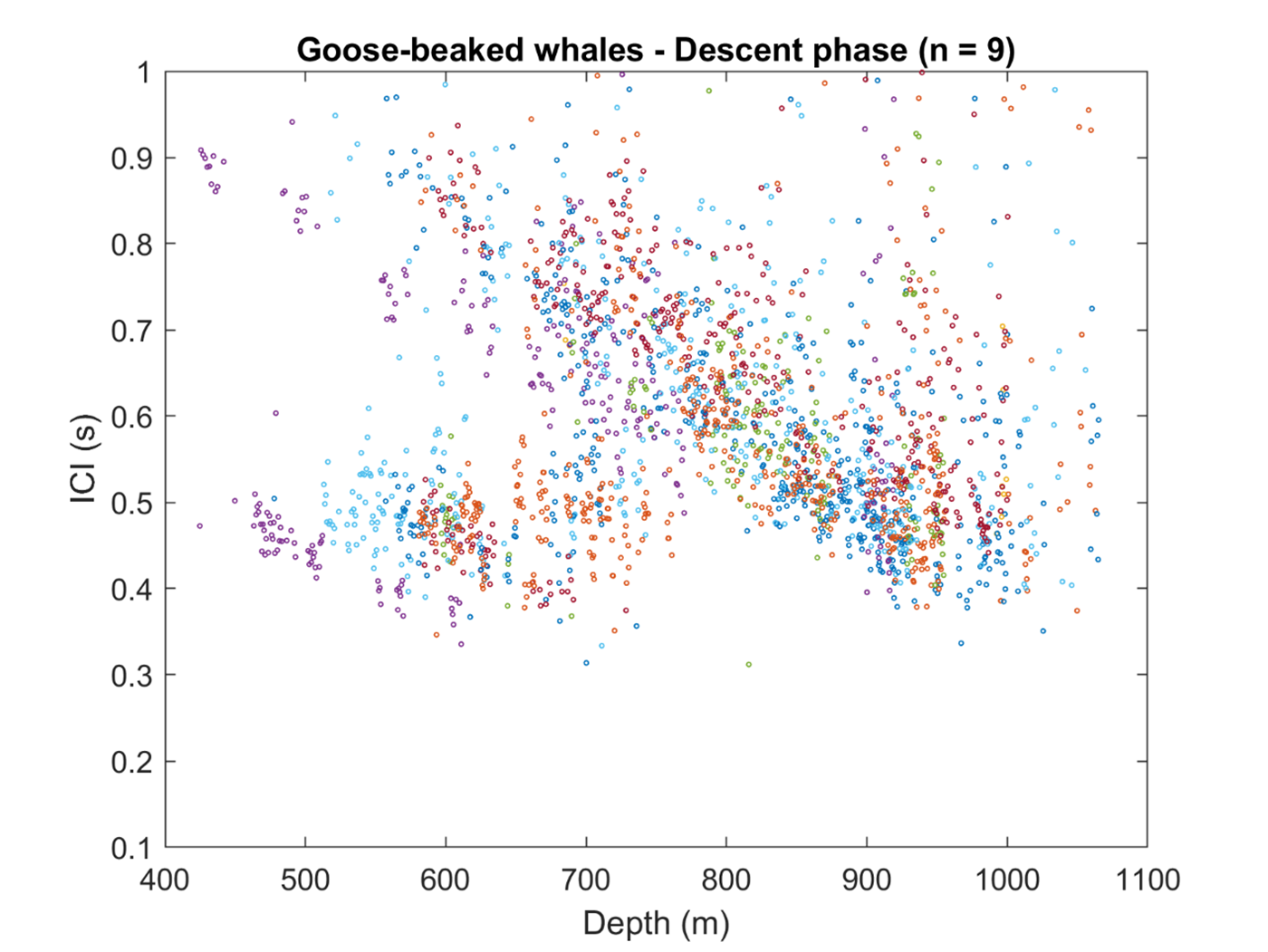

Supplement: S6 Fig — (TIF) [file pone.0340398.s006.tif]

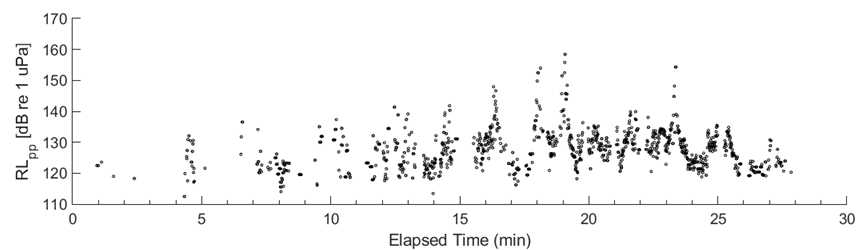

Supplement: S7 Fig — Time series plot shows short-term variability in click received levels throughout the event that are typically indicative of orientation changes with scanning behavior. (TIF) [file pone.0340398.s007.tif]

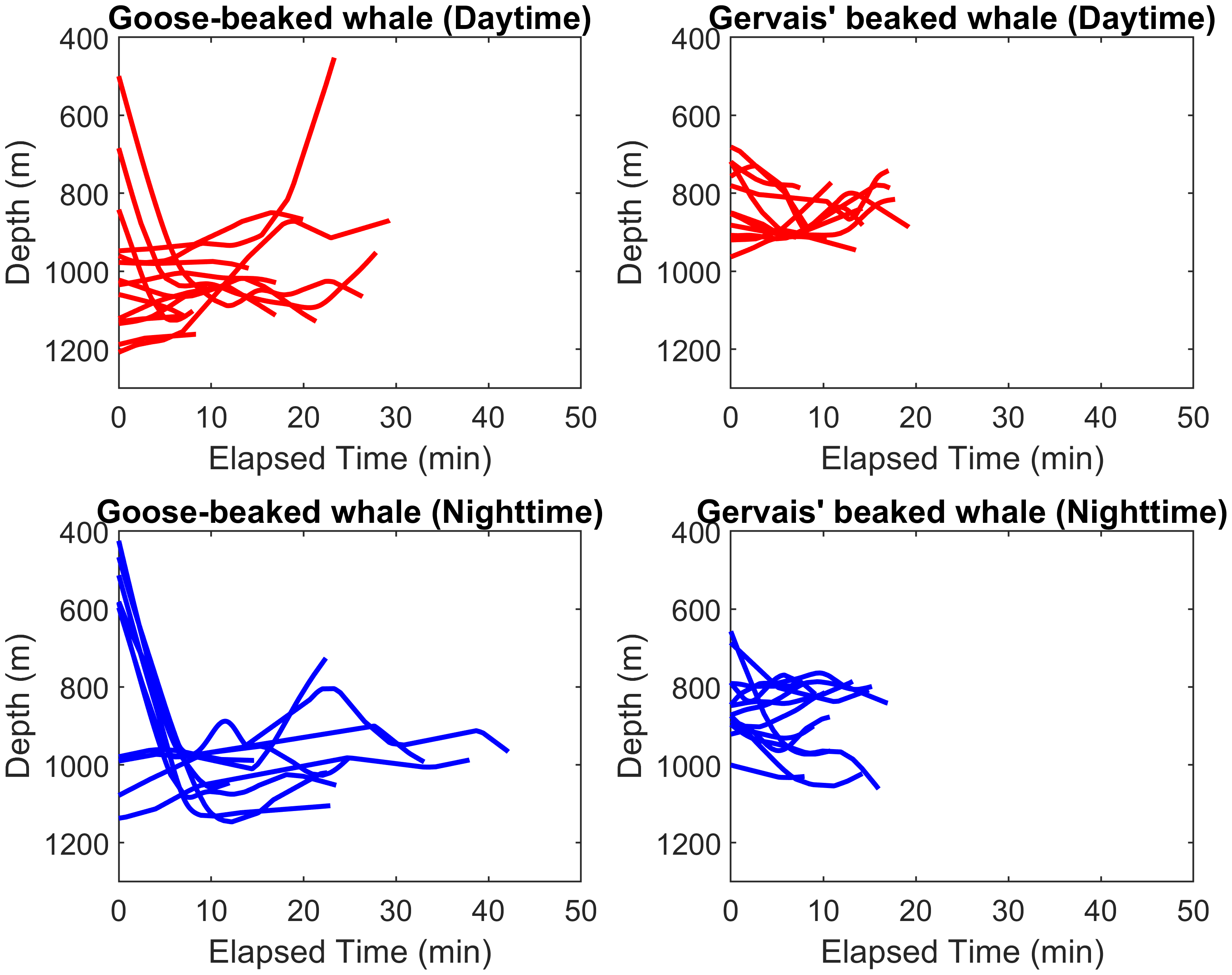

Supplement: S8 Fig — (TIF) [file pone.0340398.s008.tif]
