# Supplementary material for: Beaked whale dive behavior and acoustic detection range off Louisiana using three-dimensional acoustic tracking
Source: PLoS One. 2026 Feb 4;21(2):e0340398. doi: 10.1371/journal.pone.0340398 (PMC12871975; doi:10.1371/journal.pone.0340398)
Supplement: S1 Table — (PDF) [file pone.0340398.s009.pdf]

S1 Table. Summary statistics of dive behavior and distance estimation parameters for each of twenty-four dive tracks for goose-beaked whales detected on the GC 01 and GC 02 tracking HARPs.

	Max. horizontal distance (km)		Maximum slant distance (km)		Duration (min)	Depth (m)			Speed (m/s) / Pitch angle (°)		
Dive label	To GC 01	To GC 02	To GC 01	To GC 02		Minimum	Maximum	Mean	Descent	Bottom	Ascent
CBW1	1.23	1.09	1.24	1.09	8.4	1162	1188	1172		0.89	
CBW2	1.60	1.37	1.60	1.37	7.4	1114	1129	1118		0.55	
CBW3	0.96	0.51	0.96	0.79	26.4	500	1088	931	1.23/78.5	0.81	
CBW4	0.69	0.44	0.69	0.53	8.0	842	1125	1032	1.39/51.6	1.23	
CBW5	1.58	2.12	1.59	2.12	14.0	975	992	981		0.77	
CBW6	1.50	2.14	1.51	2.15	14.6	961	989	973		0.29	
CBW7	1.77	1.06	1.77	1.06	17.0	1038	1121	1073		1.46	
CBW8	1.66	0.96	1.66	0.96	9.6	1040	1134	1074		1.07	
CBW9	2.75	3.27	2.75	3.27	37.9	982	1137	1052		1.00	
CBW10	1.28	1.82	1.29	1.83	23.3	452	978	892		1.15	1.62/36.1
CBW11	1.58	1.71	1.59	1.73	27.8	684	1093	1052	1.20/68.3	1.26	1.34/14.4
CBW12	1.39	1.19	1.39	1.28	42.1	425	973	838	1.59/71.4	1.01	
CBW13	1.26	0.93	1.27	0.98	33.0	596	992	886	1.29/66.1	0.90	
CBW14	0.85	0.83	0.85	1.04	22.9	513	1132	865	1.57/61.9	1.03	
CBW15	0.84	0.90	0.84	0.91	23.5	584	1083	929	1.34/84.3	1.06	
CBW16	0.81	0.76	0.89	0.77	22.5	466	1146	873	1.27/68.7	1.10	
CBW17	0.57	0.51	0.78	0.65	12.0	581	1059	848	1.14/65.3	1.04	
CBW18	2.73	2.01	2.74	2.02	19.9	850	948	865		0.58	
CBW19	2.25	1.50	2.26	1.50	21.3	1018	1129	1060		0.70	
CBW20	1.34	0.57	1.34	0.58	7.0	1060	1115	1094		1.97	
CBW21	2.95	2.49	2.95	2.49	17.0	1004	1035	1019		0.97	
CBW22	2.73	2.40	2.73	2.40	24.9	952	1079	991		1.15	
CBW23	2.58	1.85	2.58	1.85	29.3	870	1208	1068		0.96	
CBW24	1.77	2.30	1.77	2.30	22.4	726	1010	904		0.74	1.24/27.8
**Average**	**1.61**	**1.45**	**1.63**	**1.49**	**20.5**	**808**	**1078**	**983**	**1.34/68.5**	**0.99**	**1.40/26.1**
**Sd**	**0.70**	**0.74**	**0.68**	**0.71**	**9.3**	**235**	**71**	**95**	**0.15/8.83**	**0.32**	**0.16/8.95**
**Min**	**0.57**	**0.44**	**0.69**	**0.53**	**7.0**	**425**	**948**	**838**	**1.14/51.6**	**0.29**	**1.24/14.4**
**Max**	**2.95**	**3.27**	**2.95**	**3.27**	**42.1**	**1162**	**1208**	**1172**	**1.59/84.3**	**1.97**	**1.62/36.2**
