# Supplementary material for: Beaked whale dive behavior and acoustic detection range off Louisiana using three-dimensional acoustic tracking
Source: PLoS One. 2026 Feb 4;21(2):e0340398. doi: 10.1371/journal.pone.0340398 (PMC12871975; doi:10.1371/journal.pone.0340398)
Supplement: S2 Table — (PDF) [file pone.0340398.s010.pdf]

S2 Table. Summary statistics of dive behavior and distance estimation parameters for each of twenty-four dive tracks for Gervais' beaked whales detected on the GC 01 and GC 02 tracking HARPs.

	Max. horizontal distance (km)		Maximum slant distance (km)		Duration (min)	Depth (m)			Speed (m/s) / Pitch angle (°)		
Dive label	To GC 01	To GC 02	To GC 01	To GC 02		Minimum	Maximum	Mean	Descent	Bottom	Ascent
GBW1	0.88	0.45	0.93	0.58	7.6	779	798	793		0.86	
GBW2	0.96	0.78	1.01	0.86	9.1	769	837	808		1.03	
GBW3	2.45	2.26	2.47	2.31	14.9	686	837	803		1.15	
GBW4	2.52	1.85	2.53	1.86	7.1	882	913	896		0.56	
GBW5	0.85	0.91	0.94	1.00	7.5	719	786	770		1.41	
GBW6	2.06	1.92	2.08	1.93	10.7	877	963	915		1.35	
GBW7	1.14	1.22	1.16	1.25	10.2	815	902	865		0.95	1.20/10.1
GBW8	1.65	1.37	1.66	1.38	13.5	911	963	927		1.25	
GBW9	0.96	0.78	1.04	0.89	10.4	718	916	862	0.93/42.5	1.09	
GBW10	2.72	2.13	2.74	2.16	17.1	742	867	795		1.11	
GBW11	0.75	0.56	0.81	0.61	19.2	730	889	773		0.81	
GBW12	0.66	0.44	0.79	0.56	17.2	681	893	793		0.82	
GBW13	1.79	1.73	1.81	1.75	17.0	764	846	789		1.02	
GBW14	1.55	0.85	1.57	0.89	15.3	786	872	810		1.02	
GBW15	1.58	0.85	1.60	0.90	13.2	786	848	831		0.99	1.11/7.55
GBW16	2.17	1.53	2.18	1.55	10.8	657	972	929	1.29/40.9	1.09	
GBW17	2.51	1.99	2.51	2.00	14.2	874	1054	1013	1.22/23.7	1.83	
GBW18	2.08	2.18	2.08	2.19	10.9	772	920	852		1.26	1.26/18.5
GBW19	2.02	2.06	2.03	2.07	14.2	835	917	882		1.04	
GBW20	1.27	0.50	1.29	0.57	17.8	815	907	857		0.92	
GBW21	1.18	1.06	1.19	1.08	8.9	900	931	921		1.19	
GBW22	2.45	1.70	2.46	1.71	16.0	896	1062	956		1.75	
GBW23	1.61	0.85	1.62	0.86	8.0	1000	1033	1026		1.44	
GBW24	1.34	1.02	1.36	1.06	14.2	838	914	885		1.17	
**Average**	**1.63**	**1.29**	**1.66**	**1.33**	**12.7**	**801**	**910**	**865**	**1.15/35.7**	**1.13**	**1.19/12.1**
**Sd**	**0.63**	**0.60**	**0.60**	**0.57**	**3.6**	**83**	**71**	**71**	**0.16/8.51**	**0.28**	**0.06/4.68**
**Min**	**0.66**	**0.44**	**0.79**	**0.56**	**7.1**	**657**	**786**	**770**	**0.93/23.7**	**0.56**	**1.11/7.55**
**Max**	**2.72**	**2.26**	**2.74**	**2.31**	**19.2**	**1000**	**1062**	**1026**	**1.29/42.5**	**1.83**	**1.26/18.5**
