# Supplementary material for: Beaked whale dive behavior and acoustic detection range off Louisiana using three-dimensional acoustic tracking
Source: PLoS One. 2026 Feb 4;21(2):e0340398. doi: 10.1371/journal.pone.0340398 (PMC12871975; doi:10.1371/journal.pone.0340398)
Supplement: S3 Table — (PDF) [file pone.0340398.s011.pdf]

S3 Table. Summary statistics of dive behavior and distance estimation parameters for each of two dive tracks for Blainville's beaked whales detected on the GC 01 and GC 02 tracking HARPs.

	Max. horizontal distance (km)		Maximum slant distance (km)		Duration (min)	Depth (m)			Speed (m/s) / Pitch angle (°)		
Dive label	To GC 01	To GC 02	To GC 01	To GC 02		Minimum	Maximum	Mean	Descent	Bottom	Ascent
BBW1	2.36	1.85	2.37	1.88	10.8	801	882	831		1.44	
BBW2	1.70	1.82	1.77	1.89	16.3	620	839	759	1.14/36.3	0.73	
**Average**	**2.03**	**1.84**	**2.07**	**1.88**	**13.6**	**711**	**861**	**795**	**1.14/36.3**	**1.09**	**NA**
**Sd**	**0.33**	**0.02**	**0.30**	**0.01**	**2.7**	**91**	**21**	**36**	**NA**	**0.36**	**NA**
**Min**	**1.70**	**1.82**	**1.77**	**1.88**	**10.8**	**620**	**839**	**759**	**NA**	**0.73**	**NA**
**Max**	**2.36**	**1.85**	**2.37**	**1.89**	**16.3**	**801**	**882**	**831**	**NA**	**1.44**	**NA**
