# Supplementary material for: Beaked whale dive behavior and acoustic detection range off Louisiana using three-dimensional acoustic tracking
Source: PLoS One. 2026 Feb 4;21(2):e0340398. doi: 10.1371/journal.pone.0340398 (PMC12871975; doi:10.1371/journal.pone.0340398)
Supplement: S4 Table — Estimates are based on 500 model iterations. (PDF) [file pone.0340398.s012.pdf]

S4 Table. Mean predicted detection rates (%) and standard deviations obtained for each beaked whale species within a 4 km radius circular area around the HARP site. Estimates are based on 500 model iterations.

**Species**	**Prediction**
Goose-beaked whale	6.8 ± 1.0
Gervais' beaked whale	3.6 ± 0.7
Blainville's beaked whale	3.2 ± 0.8
