# Supplementary material for: Beaked whale dive behavior and acoustic detection range off Louisiana using three-dimensional acoustic tracking
Source: PLoS One. 2026 Feb 4;21(2):e0340398. doi: 10.1371/journal.pone.0340398 (PMC12871975; doi:10.1371/journal.pone.0340398)
Supplement: S5 Table — (PDF) [file pone.0340398.s013.pdf]

S5 Table. Monte Carlo detectability simulation parameters by species and by study.

Simulation parameter ranges / Species	Goose-beaked whale			Gervais' beaked whale		
Study	Hildebrand et al. 2015	Li et al. 2023	This study	Hildebrand et al. 2015	Li et al. 2023	This study
Acoustic parameters						
Source Level Mean (dBpp)	225	225	223–228	220	220	215–220
Source Level Std. Dev. (dBpp)	3		1–4	3		1–2
Peak Frequency (kHz)	40	40.2	40	43.8	43.8	40
Minimum off-axis amplitude loss (dBpp) - Side			28–32			28–32
Minimum off-axis amplitude loss (dBpp) - Back			28–32			38–42
Directivity Index	24–28	Piston model with radius of 16 cm	24–28	24–28	Piston model with radius of 16 cm	18–22
Dive parameters						
Depth at Start of Clicking Mean (m)		750	450–460		750	420–430
Depth at Start of Clicking Std. Dev. (m)		50	60–80		50	20–30
Dive Depth Mean (m)		1100	980–990		1100	860–870
Dive Depth Std. Dev. (m)		50	90–100		50	60–80
Dive Altitude Mean (m)	175–225	50-100	100–110	175–225	50-100	200–300
Dive Altitude Std. Dev. (m)			50–60			50–100
Dive Depth Max (m)			3,000			3,000
Descent Angle Mean (degrees)		Beta between −90° and 0° with a = 2, b = 5	70–75		Beta between −90° and 0° with a = 2, b = 5	35–40
Descent Angle Std. Dev. (degrees)			5–10			5–10
Maximum model radius (km)	4	10	4	4	10	4
Number of points simulated	10,000	100,000	100,000	10,000	100,000	100,000
